# Time delays shape the eco-evolutionary dynamics of cooperation

**DOI:** 10.1038/s41598-023-41519-1

**Published:** 2023-08-31

**Authors:** Sourav Roy, Sayantan Nag Chowdhury, Srilena Kundu, Gourab Kumar Sar, Jeet Banerjee, Biswambhar Rakshit, Prakash Chandra Mali, Matjaž Perc, Dibakar Ghosh

**Affiliations:** 1 Department of Mathematics, Jadavpur University, Kolkata, 700032 India; 2 Department of Environmental Science and Policy, University of California, Davis, CA 95616 USA; 3 Department of Ecology & Evolution, University of Chicago, Chicago, IL 60637 USA; 4 Physics and Applied Mathematics Unit, Indian Statistical Institute, Kolkata, 700108 India; 5 BYJU’S, Think & Learn Pvt. Ltd., IBC Knowledge Park, 4/1 Bannerghatta Main Road, Bangalore, 560029 India; 6 Department of Mathematics, Amrita School of Physical Sciences, Amrita Vishwa Vidyapeetham, Coimbatore, 641112 India; 7 Faculty of Natural Sciences and Mathematics, University of Maribor, Koroška cesta 160, 2000 Maribor, Slovenia; 8 Department of Medical Research, China Medical University Hospital, China Medical University, Taichung, 404332 Taiwan; 9 Alma Mater Europaea, Slovenska ulica 17, 2000 Maribor, Slovenia; 10 Complexity Science Hub Vienna, Josefstädterstraße 39, 1080 Vienna, Austria; 11 Department of Physics, Kyung Hee University, 26 Kyungheedae-ro, Dongdaemun-gu, Seoul, Republic of Korea

**Keywords:** Complex networks, Nonlinear phenomena

## Abstract

We study the intricate interplay between ecological and evolutionary processes through the lens of the prisoner’s dilemma game. But while previous studies on cooperation amongst selfish individuals often assume instantaneous interactions, we take into consideration delays to investigate how these might affect the causes underlying prosocial behavior. Through analytical calculations and numerical simulations, we demonstrate that delays can lead to oscillations, and by incorporating also the ecological variable of altruistic free space and the evolutionary strategy of punishment, we explore how these factors impact population and community dynamics. Depending on the parameter values and the initial fraction of each strategy, the studied eco-evolutionary model can mimic a cyclic dominance system and even exhibit chaotic behavior, thereby highlighting the importance of complex dynamics for the effective management and conservation of ecological communities. Our research thus contributes to the broader understanding of group decision-making and the emergence of moral behavior in multidimensional social systems.

## Introduction

The prisoner’s dilemma game^1,2^ is perhaps the most well-known and widely studied toy model analyzing the conflict between individual and collective interests^3^ among self-interested individuals. Selfish people always aim to maximize their well-being, which drives a formidable challenge to the evolution of cooperative behavior. Researchers remain curious to identify the necessary conditions and underlying mechanisms to obtain cooperation by natural selection. The prisoner’s dilemma game seems the best suited to address the subtleties of cooperation within groups of selfish individuals. The original version of this one-shot game consists of two rational individuals who have to decide independently to each other whether to defect or cooperate. Those selfish players’ curiosity lies in maximizing their payoff, so they always opt for defection, which promises a better return irrespective of the opponent’s chosen strategy in the classical prisoner’s dilemma game. Consequently, this mutual defection impedes the formation of a cooperative state, leading to the tragedy of the commons^4^, and hence, the society suffers. How to overcome this unfavorable outcome, often at odds with reality, is a basic query of the evolutionary game dynamics and evolutionary biology.

The cooperation of simpler parts^5^ helps in evolutionary progress. The formation of the genome from genes, multicellular organisms from single cells, helper birds assisting their parents in feeding their young^6^, and many more are excellent examples of cooperative enterprise. Social structures like groups and societies are examples of collaborative efforts by individuals despite cooperation always being vulnerable to exploitation by defectors. In spite of nobody knowing everything, human beings can still survive, thanks to their cooperative attitude. The large-scale collaboration among humans leads to the formation of towns, cities, states, countries, and continents. All these collective behaviors urge to determine mechanisms enforcing the appearance of cooperation in societies. A plethora of research has been conducted in the last decade recognizing several cooperation-facilitating mechanisms. The pivotal studies of Refs.^7–9^ show how nearest-neighbor interactions in spatial structure can help to sustain cooperation in iterated prisoner’s dilemma games, depending on the temptation to defect. The vital role of network topologies in flourishing a cooperative state^10–12^ has been a subject of rigorous investigation since the development of the discipline of network science. The mobility of the players^13^, kin selection^14^, reproduction restrictions^15^, impact of aging^16^ and direct and indirect reciprocity^17,18^ are all found to be highly potent promoters of cooperative behavior in the spatial prisoner’s dilemma game. People often introduce an additional strategy along with unconditional cooperation and defection. Introducing a tit-for-tat strategy^19^ is one of the most successful strategies for the iterated prisoner’s dilemma game for entailing cooperation. Various studies also confirm the positive role of punishment^20–28^ in elevating collaborative efforts in our society.

Recently, Nag Chowdhury et al.^29^ examined the impact of punishment on the prisoner’s dilemma game in the presence of the altruistic free space. Interestingly, although the influence of free space on emergent collective behavior^30–36^ gains well-deserved attention among interdisciplinary researchers, its generous contribution is often neglected from the game’s theoretical point of view. Reference^29^ considers the self-sacrificing contribution of free space by allowing others to replicate at the expense of its fitness. In return, free space never expects any favor from others. This inclusion of the natural instinct of free space allows them to consider an eco-evolutionary model where ecological changes and species evolution co-occur. The earlier assumption^37^ that evolutionary processes are much slower than ecological processes is relaxed in Ref.^29^. Numerous investigations^38–43^ have recently been conducted to understand the reciprocal effects of evolution and ecology. To understand how evolution influences ecology and how ecology affects evolution, scientists began studying the interaction between evolution and ecology on the same timescale^44–51^. The concept of co-evolution has also been considered in terms to depict the interactions between homogeneous and heterogeneous strategies taken by the player kinds those get useful in their evolution in the society. As ecological free space plays pivotal role in order to shower benefits in the evolution of the player populations having different actions for the dilemma, this kind of co-evolution can also be identified as eco-evolution in our study.

Motivated by all these studies, we wish to investigate the influence of time delays on the evolution of cooperation in the prisoner’s dilemma game by introducing additional strategies like punishment and altruistic free space. Most of the previous studies on evolutionary game dynamics assume the interactions among rational players are instantaneous. Interestingly, the significance of finite time propagation delay is enjoying widespread recognition in interdisciplinary research due to its numerous practical applications^52–55^. A variety of natural systems requires a finite time propagation delay to materialize. The echoes in an auditorium^56^, the chirping of crickets^57^, the information flow in the nervous system^58^, and many other examples naturally involve a time delay parameter. Time delay is also ubiquitous in many biological processes, such as digestion^59^, maturation^60,61^, incubation^62^, and feedback^63^. How delay can affect collective behaviors in coupled dynamical systems^64–72^ has been investigated comprehensively. Recently, Ref.^73^ demonstrates the complex replicator dynamics of a delayed multigame with environmental space. How delayed distribution of continuously accumulating goods affects the evolution of cooperation in the spatial public goods games on the square lattice is revealed in Ref.^74^. Li et al.^75^ explore the bifurcation control of a fractional-order Lotka-Volterra model using delay feedback control. The profound impact of time delay on the stability of the interior equilibrium point of the pure strategy model is addressed in Ref.^76^. An exciting finding of Ref.^77^ uncovers that delay applied to the interspecific interactions can only affect the convergence time of the cooperation rate; however, the stability of the equilibrium points remains unaltered. The scenario differs in the case of delayed intraspecific interactions, where the system oscillates around the equilibrium point as the time delay period increases. Ben-Khalifa et al.^78^ inspect the stability of the evolutionarily stable strategy in the continuous-time replicator dynamics with random time delays.

All these pioneering works inspire us to study the role of time delay in spreading cooperative clusters among rational individuals. To understand how cooperation prevails in a world of selfish individuals, we consider a delayed eco-evolutionary model where free space provides unselfish benefits to all others. We update the evolutionary dynamics of the prisoner’s dilemma game by incorporating the effect of punishment and free space. The influence of time delay on the eco-evolutionary dynamics being a relatively unaddressed problem, we analyze this issue using the delayed eco-evolutionary model aimed towards resolving the dilemma raised in the prisoner’s dilemma game.

## Model

We start with the evolutionary two-strategy weak version of the prisoner’s dilemma game, where players can decide whether to cooperate $(\textbf{C})$ or defect $(\textbf{D})$. For mutual cooperation, they both can earn a reward *R*. The mutual defection yields both the player punishment *P*. The dealing between a cooperator and a defector gives the sucker’s payoff *S* to the cooperator, while the defector receives the temptation to defect *T*. Throughout our investigation, we maintain the ranking between the payoff as $T>R>P\ge S$ and adopt the same parameter values $T=\beta >1$, $R=1$, and $P=S=0$ from Refs.^10,29^. This inequality of the weak prisoner’s dilemma game indicates that mutual defection always promises less payoff, as $0=P<R=1$. However, from the individual standpoint, defection serves as the intelligent strategy between the two competing strategies. If the player chooses cooperation, the defector earns more as $\beta =T>R=1$. If the opponent also decides to defect, still the defector can not earn more by cooperating due to our choice of parameter values. The $2 \times 2$ payoff matrix looks like1in which the entries represent the payoff accumulated by the player in the left. We consider an additional strategy, the ‘punisher’ who acts like a cooperator and receives the same payoff $R=1$ if the other player decides to cooperate. However, if the opponent player defects, the punisher will use their own resources to punish them and earn a payoff value of $S-\delta =-\delta$. In return, the defector makes $T-\delta =\beta -\delta$ with $\delta >0$. Note that a defector earns more if they interact with a cooperator. This inclusion of punishers will extend our payoff matrix (1) to the following matrix2

We further incorporate the ecological contribution of free space in the payoff matrix. Free space allows others to replicate and provide others ample opportunity to survive. Interestingly, free space never anticipates anything in return, and this selfless contribution of free space motivates us to update our payoff matrix (2) in the following way,3

From the above upgraded payoff matrix (3) and in evolutionary sense, ecological free space (*F*) seems out to be one selfless strategy to be made by any player population and on interaction with this, each population kind gets positive attributes as their benefits for replication, whereas, free space stands out to exist independently for every player kinds to shower it’s altruistic ease, without being chosen by any trait as their action in evolution. In order to showcase our dynamical system, we use the positive attributes as benefits to the competing players from this spatial free space but in game theoretic sense, free space does not act as an evolutionary move. Here, all these parameters $\sigma _1$, $\sigma _2$, and $\sigma _3$ are strictly positive quantities, as free space contributes altruistically to all the rational individuals. Free space earns only zero in return as it never expects any benefits for its generous acts. Now we calculate each strategy’s fitness and determine the evolution of populations by assuming that an individual’s reproduction rate depends solely on their average fitness. Let us assume *x*, *y*, *z*, and *w* be the respective fraction of cooperators, punishers, defectors, and free space, respectively. Hence, $x,y,z,w \in [0,1]$ and $x+y+z+w=1$. Interestingly, the process of learning takes time and effort. It takes a considerable amount of time for people to adapt strategies based on the information they learn. The players need to gather the information at each round of the game and then assess the effectiveness of the methods. Based on their understanding, they spread that information which helps others to recognize which strategy is the most successful in society. Thus individuals select a strategy at time *t* based on the fitness before $\tau \ge 0$ time instance. We introduce the variables $x_\tau =x(t-\tau )$, $y_\tau =y(t-\tau )$, $z_\tau =z(t-\tau )$ and $w_\tau =w(t-\tau )$. Since we are interested in inferring the obtained results using fundamental principles of biological systems, we maintain the constraint $x_\tau + y_\tau +z_\tau +w_\tau =1$ along with $x_\tau ,y_\tau ,z_\tau ,w_\tau \in [0,1]$ throughout the article. This constraint helps us to eliminate one independent variable and construct a simple eco-evolutionary model. The respective average fitness of cooperators, punishers, defectors and free space is given by

4 $$\begin{aligned} f_\textbf{C}&=\, x_\tau + y_\tau +\sigma _{1}w_\tau =(1-\sigma _{1})x_\tau + (1-\sigma _{1})y_\tau -\sigma _{1}z_\tau +\sigma _{1}, \nonumber \\ f_\textbf{P}&=\, x_\tau +y_\tau -\delta z_\tau +\sigma _{2}w_\tau =(1-\sigma _{2})x_\tau + (1-\sigma _{2})y_\tau -(\delta +\sigma _{2})z_\tau +\sigma _{2}, \nonumber \\ f_\textbf{D}&=\, \beta x_\tau +(\beta -\delta )y_\tau +\sigma _{3}w_\tau =(\beta -\sigma _{3}) x_\tau +(\beta -\delta -\sigma _{3})y_\tau -\sigma _{3}z_\tau +\sigma _{3},\nonumber \\ f_\textbf{F}&=\, 0. \end{aligned}$$

We assume all players die with a uniform rate $\xi >0$. Thus, our proposed delayed system looks like

5 $$\begin{aligned} \dot{x}=&\, {} x[f_\textbf{C}-\xi ],\nonumber \\ \dot{y}=&\, {} y[f_\textbf{P}-\xi ],\nonumber \\ \dot{z}=&\, {} z[f_\textbf{D}-\xi ]. \end{aligned}$$

Thus substituting Eq. (4) in Eq. (5), we obtain

6 $$\begin{aligned} \dot{x}=&\, {} x[(1-\sigma _{1})x_\tau + (1-\sigma _{1})y_\tau -\sigma _{1}z_\tau +(\sigma _{1}-\xi )],\nonumber \\ \dot{y}=& \, {} y[(1-\sigma _{2})x_\tau + (1-\sigma _{2})y_\tau -(\delta +\sigma _{2})z_\tau +(\sigma _{2}-\xi )],\nonumber \\ \dot{z}=& \, {} z[(\beta -\sigma _{3}) x_\tau +(\beta -\delta -\sigma _{3})y_\tau -\sigma _{3}z_\tau +(\sigma _{3}-\xi )]. \end{aligned}$$

Note that $\tau =0$ gives the non-delayed system. In the subsequent section, we comprehensively discuss the difference between the outcomes in delayed and non-delayed systems.

## Numerical results

### Comparison between delayed and non-delayed model

**Figure 1:**
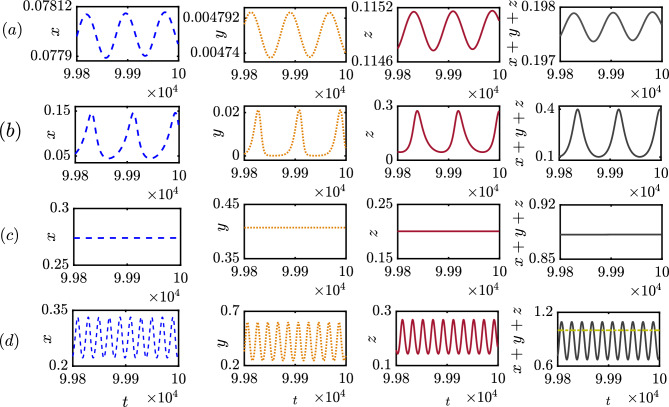
A comparison of time series between delay-free and delayed systems: The time series in this figure compares the dynamics of the system (6) with and without delay. (**a**) and (**c**) Correspond to the dynamics of the non-delayed system for $\tau =0$, that displays either a small-amplitude oscillation in its three variables’ temporal evolution (**a**) or convergence to the interior equilibrium (**c**) owing to the choice of different parameter values. In contrast, the inclusion of delay generates larger oscillations in both cases, corresponding time series are displayed in (**b**) and (**d**) for chosen values of the delay parameter $\tau = 0.24$ and $\tau = 1.5$, respectively. Temporal evolution of population fraction of the cooperators, punishers and defectors are depicted in the first, second and third columns, respectively, while the evolution of the total population ($x + y +z$) is shown in the last column. Non-zero delay may trigger the total population fraction to cross the upper bound of unity, leading to an overcrowded solution as shown in the last panel. The coexistence of all three populations either in oscillation or in equilibrium helps maintaining the biodiversity. The initial conditions for the non-delayed variables are set at (0.3, 0.3, 0.3), while those for the delayed state variables are set at (0.25, 0.25, 0.25) in all subfigures. The parameter values are fixed at $\xi = 0.50$, $\beta = 2.15$, $\delta = 1.4$, $\sigma _{1} = 0.52$, $\sigma _{2} = 0.72$ and $\sigma _{3} = 0.41$ for panels (a-b), and $\xi = 0.80$, $\beta = 1.25$, $\delta = 0.30$, $\sigma _{1} = 1.00$, $\sigma _{2} = 1.50$ and $\sigma _{3} = 0.60$ for panels (**c**,**d**).

To understand the impact of time delay in population dynamics, specifically in the context of a prisoner’s dilemma game, we present the comparative temporal behavior of variables in the presence and absence of delay in Fig. 1. To start with, we consider the values of the parameters as $\xi = 0.50$, $\beta = 2.15$, $\delta = 1.4$, $\sigma _{1} = 0.52$, $\sigma _{2} = 0.72$ and $\sigma _{3} = 0.41$ in the subfigures (a,b). Subfigures of (a) demonstrate small amplitude oscillations of all variables without delay. Clearly, the chosen parameter values help the defectors to dominate others as we identify the inequality $z>x>y$. It should be noted that our observation may alter for a different set of initial conditions, as the system (6) is multistable. Later, we will examine the role of initial conditions in our model in detail. We fix the initial conditions at $(x_0, y_0, z_0)=(0.3, 0.3, 0.3)$. We further set the initial conditions for the delay variables as $x_\tau (0)=0.25, y_\tau (0) = 0.25$, and $z_\tau (0) = 0.25$ for the subfigures in the presence of delay. The chosen parameter values and initial conditions provide an opportunity to maintain biodiversity by allowing the coexistence of all strategies in subfigures (a,b). The range of oscillations for $x \in [0.0775,0.0779]$, $y \in [0.004732, 0.00477]$, and $z \in [0.1145, 0.1148]$ is petite in subfigures (a), where the sum of the population lies within the range [0.197, 0.1975]. Since the punishment parameter’s value $(\delta =1.4)$ is high enough, punishers can not afford to survive in the long run, despite the free space-induced benefits towards the punishers being higher as per our chosen parameter values $(\sigma _2>\sigma _1>\sigma _3)$. Since the temptation to defect is high for the defectors as $\beta =2.15$, defectors are able to overcome the hurdle in the long run in our model. We further analytically calculate the interior equilibrium point (0.0777, 0.0047, 0.1147)) for this set of parameter values, which is found to be an unstable focus node as the eigenvalues of the Jacobian at this point are $\lambda _1=0.1236$, and $\lambda _{2,3}=-0.004 \pm 0.0785i$ where $i=\sqrt{-1}$. To investigate the delay effect, we allow a small amount of delay $\tau =0.24$ in the state variables in subfigures (b) by keeping fixed all the parameters’ values and initial conditions of subfigures (a). This inclusion of delay will not alter the inequality $z>x>y$ observed in subfigures (a); however, the amplitude of oscillations amplifies. We find $x \in [0.0447, 0.1462]$, $y \in [0.0001126, 0.02117]$, and $z \in [0.04503, 0.2736]$ in subfigures (b) and their overall sum remains bounded in [0, 1].

A striking difference is also observed for the subfigures (c,d) with the fixed parameter values $\xi = 0.80$, $\beta =1.25$, $\delta = 0.30$, $\sigma _{1} = 1.00$, $\sigma _{2} =1.50$, and $\sigma _{3} =0.60$ and fixed initial conditions $x_0=0.3$, $y_0=0.3$ and, $z_0=0.3$. The subfigure is drawn additionally with the delay parameter $\tau = 1.5$ with fixed initial condition $x_\tau (0)=0.25, y_\tau (0)=0.25$, and $z_\tau (0)=0.25$. Subfigure (c) reveals the coexistence of all strategies, and we analytically calculate the interior equilibrium (0.274, 0.407, 0.2) for the chosen parameter values. We find this steady state is a locally stable focus node as the eigenvalues of the Jacobian at this steady state are $\lambda _1=-0.0208$, $\lambda _{2,3}=-0.3327 \pm 0.2251i$. Interestingly, the punishers’ population is the dominant in this case as we identify the inequality $y>x>z$. The lower abundance of defectors is due to the choice of insufficient temptation to defect $(\beta =1.25)$ and free space-induced benefits towards defectors $\sigma _{3} =0.60$ is lower. Punishers overcome the fierce struggle as punishment parameter value $\delta = 0.30$ is chosen sufficiently low. Punishers use their own resources to penalize the defectors, and since $\delta$ is chosen low here, punishers’ resources are not overly utilized. Furthermore, our selected parameter values suggest free space-induced benefits toward punishers are higher compared to others $(\sigma _2>\sigma _1>\sigma _3)$. The addition of a time delay of suitable strength not only destroys the stability of this interior point but also yields an overcrowded solution as $x+y+z$ exceeds 1. This introduction of time delay leads to an instantaneous change in the system’s dynamics.

The emergence of these oscillations hints us the spontaneous emergence of cyclic dominance among those strategies. In the following subsection, we discuss this occurrence in more detail.

### Cyclic dominance

**Figure 2:**
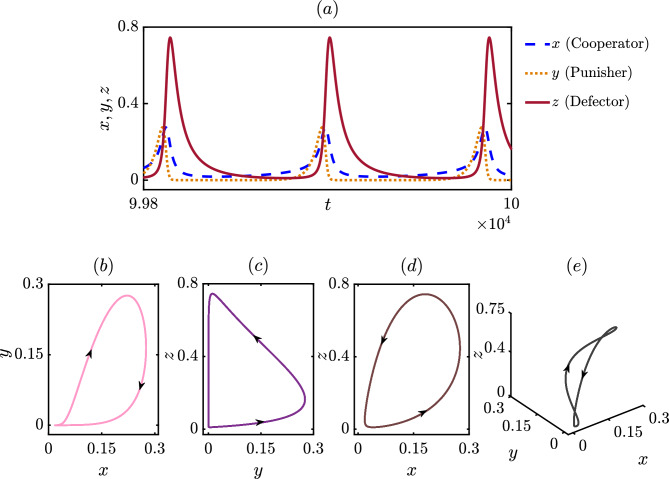
Cyclic dominance among three competing strategies: (**a**) Temporal dynamics of the oscillatory coexistence of all three strategies exhibiting the emergence of cyclic dominance among the species that allows each speices to dominate others. The following parameter values are chosen for numerical simulation: $\xi = 0.5$, $\beta = 2.5$, $\delta = 1.39$, $\sigma _{1} = 0.52$, $\sigma _{2} = 0.72$, $\sigma _{3} = 0.41$ and, $\tau = 0.019$. Higher value of the temptation parameter $\beta$ facilitates the defectors although the punishment parameter $\delta$ helps controlling their population by penalizing them. On the other hand cooperators get more benefit from the altruistic free space compared to the defectors due to our chosen parameter values, which helps in maintaining the coexistence of all three strategies. Phase portraits of the periodic attractor along with the direction of the flow in (**b**–**d**) two dimensions and in (**e**) three dimension. The initial values for the state variables associated with the player strategies are considered to be, $(x_0,y_0,z_0) = (0.3,0.3,0.3)$ and those for the delayed variables, are considered to be, $(x_\tau (0),y_\tau (0),z_\tau (0)) = (0.25,0.25,0.25)$.

We consider the same parameter values $\xi =0.50$, $\beta = 2.5$, $\delta = 1.39$, $\sigma _1=0.52$, $\sigma _2=0.72$, $\sigma _3= 0.41$ and $\tau =0.019$ and integrate our model (6) using Huen’s method^79^ with $10^7$ number of iterations. After discarding a sufficiently long transient of length $9.9 \times 10^6$ iterations, we present their delayed eco-evolutionary dynamics in Fig. 2. We maintain the same initial conditions as in Fig. 1b. Interestingly, the delay parameter helps the system maintain oscillatory dynamics that attest to the emergence of cyclic dominance in our model. Cyclic dominance^80^ allows each strategy to dominate others for a specific time window, which is impossible if we attain steady-state dynamics. The periodic dynamics of each variable portray the coexistence of competing strategies. The maxima of each variable in the first row in Fig. 2 unveils $z_{max}>x_{max}>y_{max}$. Since our chosen value of the temptation parameter, $\beta =2.50$ is higher than the payoffs of a cooperator and punisher, $z_{max}$ can attain such larger values in its temporal dynamics. On the other hand, the punishment parameter, $\delta$, is fixed at 1.39, which helps to control the defection by penalizing them; however, the punisher also loses this amount while punishing the defectors. This makes punishers vulnerable in society for our specific choices of initial conditions and parameter values. Furthermore, the interplay between all parameters is more pronounced in this figure, as despite altruistic free space contributing more towards the punishers and lesser towards the defectors $(\sigma _2>\sigma _1>\sigma _3)$, punishers are still unable to take over the defectors. The temptation to defect is so high that it helps to ignore the selfless contribution of free space, and thus defectors can gain a higher density in the long run. Nevertheless, periodic oscillation allows a window of opportunity for the cooperators to invade the defectors, who in turn can invade the punishers and, ultimately, punishers are able to overrun the cooperators. In this way, cyclic dominance emerges spontaneously and captures the beauty of governing eco-evolutionary dynamics. We further confirm that the obtained solution and their sum remain bounded within [0, 1], providing a biologically feasible solution. The two and three-dimensional projections of the attractor, along with the direction of the flow, are plotted in the second row of Fig. 2.

In the next subsection, we will scrutinize the underlying mechanism that drives the system toward an oscillatory behavior.

### Exploring the role of delay in the eco-evolutionary dynamics

**Figure 3:**
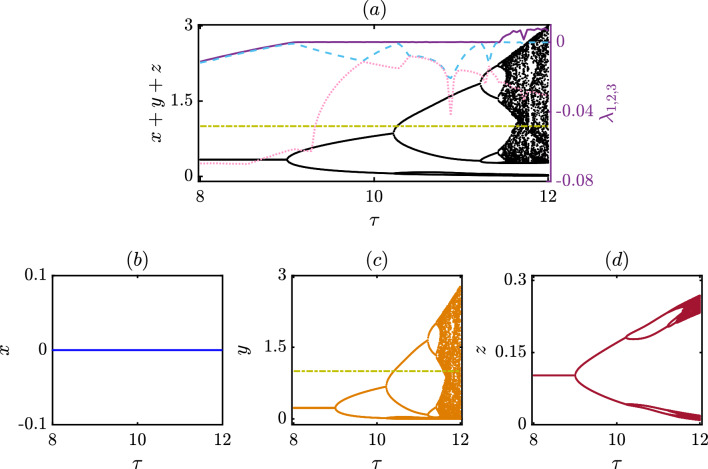
Bifurcation diagram and the Lyapunov exponents of the proposed delay model: (**a**) The effect of the delay parameter $\tau$ on the dynamics of the total population $x+y+z$ of all three species, that indicates the emergence of periodic oscillation via Hopf bifurcation at $\tau = 8.99$ which then leads to period doubling bifurcation as delay increases and eventually to chaos. The three Lyapunov exponents $\lambda _{1, 2, 3}$ of the delayed system are also displayed among which $\lambda _1$ (solid purple) is the maximum that validates whether the dynamics is a steady state, periodic or chaotic oscillation contingent to its value being negative, zero or positive, respectively. The second largest exponent $\lambda _2$ (cyan dashed) remains negative throughout the delay interval except at the bifurcation points where it takes zero value and $\lambda _3$ (pink dotted) remains negative throughout the interval. The individual bifurcation diagrams are also shown in (**b**) for cooperators *x*, (**c**) for punishers *y*, and (**d**) for defectors *z*, respectively. The following parameter values are chosen: $\xi$ = 1.20, $\beta$ = 1.60, $\delta$ = 0.30, $\sigma _{1}$ = 1.35, $\sigma _{2}$ = 1.50, $\sigma _{3}$ = 1.35 and the initial conditions are set at (0.1, 0.2, 0.5) for the non-delayed variables and (0.3, 0.3, 0.3) for the delyaed variables. The figures are plotted by taking the $\tau$ step length of 0.005 for the bifurcation diagrams and 0.04 for the Lyapunov exponents in the range [8, 12]. From the individual bifurcation diagrams it is clear that the chosen parameter values correspond to the coexistence of only the punishers and defectors, with punishers being the dominating population.

To further study the effect of delay in our model (6), we keep all parameter values fixed at $\xi = 1.2$, $\beta = 1.6$, $\delta = 0.30$, $\sigma _{1} = 1.35$, $\sigma _{2}= 1.50$, $\sigma _{3} = 1.35$ and vary the delay parameter $\tau$ within the closed interval [8, 12] with a fixed step length 0.005 and plot the bifurcation diagrams in Fig. 3. We find out the cooperators extinct in the long run throughout the interval, as *x* remains at zero in Fig. 3b. Nevertheless, the dynamics of the punishers and defectors offer a great variety of dynamical behaviors. Our system experiences a series of transitions from a periodic to a chaotic state as $\tau$ is varied in Fig. 3. All these figures provide valuable insight into how our system responds to changes in the delay parameter.

We identify punishers overrule the defectors in the steady state regime for the chosen initial conditions and parameter values. Assuming an initial condition of (0.1, 0.2, 0.5) for the state variables and (0.3, 0.3, 0.3) for the delayed variables, we performed $10^7$ iterations of the system (6). We discarded the first $9.8 \times 10^6$ iterations to ensure that our analysis focuses only on the system’s long-term behavior. However, this steady state loses its stability and gives rise to a periodic solution beyond a specific value of $\tau$. We will provide a thorough analysis later to detect the point of this Hopf bifurcation analytically. Interestingly, the range of *y* is vast compared to that of *z* (c.f. subfigures (c,d) of Fig. 3). This indicates the punishers gain some kind of opportunity through our setup and can dominate the defectors. Notably, the free space-induced benefits towards the punishers are slightly larger than others $(\sigma _{2}>\sigma _{1}=\sigma _{3})$ in this figure. Interestingly, we observe this periodic solution loses its stability and gives rise to a new periodic solution with twice the period of the original one as the delay parameter value is increased. As $\tau$ is further increased, the system goes through additional period-doubling bifurcations, giving rise to periodic solutions with four times, eight times, and so on, the period of the original periodic attractor. Eventually, the system enters a chaotic regime, where the dynamics are unpredictable and sensitive to small perturbations. To further validate our findings, we use the Lyapunov exponent to measure the sensitivity of our eco-evolutionary model (6) to small perturbations in its initial conditions. The calculation of Lyapunov exponents for delayed systems is generally more complicated than that of non-delayed systems due to the need to consider the effect of time delay on the system’s dynamics. We calculate the Lyapunov exponents of the system for $\tau \in [8,12]$ by increasing $\tau$ with a fixed step length 0.04. Since our system (6) contains only one delay parameter, the number of Lyapunov exponents is equal to the dimensionality of the non-delayed system (i.e., the number of variables in the system). We plot each of these Lyapunov exponents in Fig. 3a.

**Figure 4:**
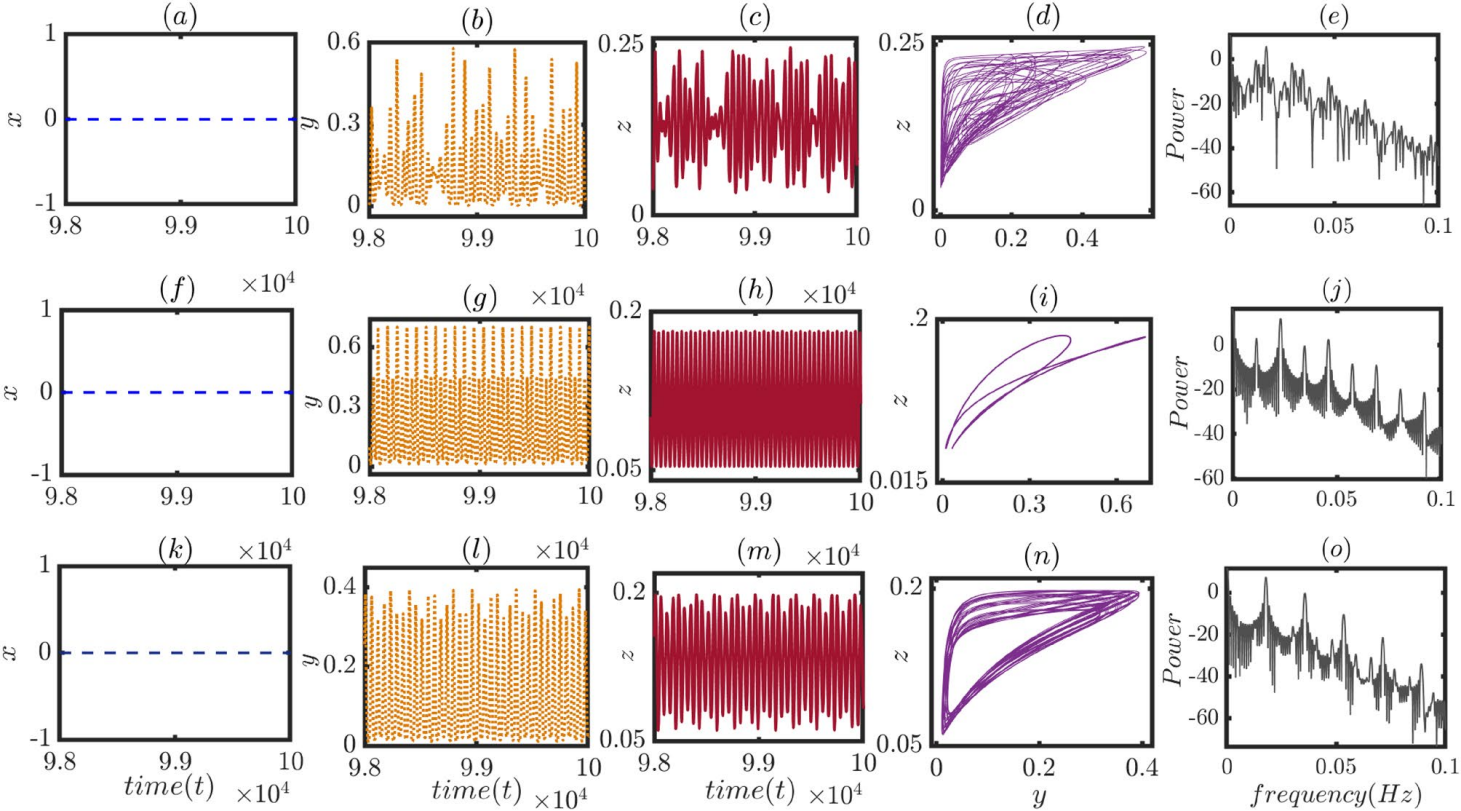
Different dynamical structures of the player populations along with their power spectra: The effect of the temptation parameter and delay parameter on the temporal dynamics of the delayed system are displayed for three different choices of $(\beta , \tau )$ values in three different panels. Time series of the cooperators, punishers and defectors are portrayed in the first, second and third columns, respectively. The fourth column corresponds to the trajectory in the $y-z$ parameter space, and finally, in last column the power spectra using fast Fourier transformation is presented for three different temporal dynamics. First panel (**a**–**e**) exhibits the chaotic dynamical behavior in *y* and *z* variables for the chosen parameter values $(\beta , \tau ) = (1.93, 11.64)$, second panel (**f**–**j**) corresponds to the emergence of two periodic oscillation in punishers’ and defectors’ dynamics for the chosen parameter values $(\beta , \tau ) = (1.64, 10.73)$, and third panel (**k**–**o**) displays the quasi-periodic dynamics among the punisher and defector populations with irregular periodicity for the parameter values $(\beta , \tau ) = (1.90, 10.50)$. In all three cases, the cooperators remain extinct due to the choice of the other parameter values which are fixed at $\xi = 1.2, \delta = 0.3, \sigma _1 = 1.35, \sigma _2 = 1.5, \sigma _3 = 1.35$. These three distinct dynamical states are also justified by the power spectrum analysis that unveils the nature of the peaks of the power spectra measured with respect to the normalized frequency. The initial values of the population fractions are taken to be (0.1, 0.2, 0.5) for the non-delayed variables, and (0.3, 0.3, 0.3) for the delayed variables. We have observed the resultant dynamics after wiping out the initial $98 \times 10^{5}$ out of the $10^7$ numbers of iterations.

The first and the second largest Lyapunov exponents reveal valuable information about our system. The largest Lyapunov exponent $\lambda _1$, shown in deep purple, remains negative up to a certain value of $\tau$, indicating stable steady states. For a suitable range of the delay parameter $\tau$, it converges to zero, which reflects a standard signature of having a periodic solution in our system (6). In both of these stable states, nearby trajectories converge towards each other, meaning that small perturbations in the system’s initial conditions lead to similar outcomes. Beyond this range of $\tau$, it offers only the positive value describing the rate of exponential divergence of nearby trajectories. This positive Lyapunov exponent $\lambda _1$ identifies the onset of chaos and validates our bifurcation diagram, which reflects the period doubling route to chaos. Interestingly, most of the previous studies on Lyapunov exponents only concentrate on the largest Lyapunov exponent and ignore the possibility of extracting invaluable information from the other Lyapunov exponents. The second-largest Lyapunov exponent $\lambda _2$, shown in cyan color dashed line in Fig. 3a, discloses fascinating details on the bifurcation point in our study. It initially remains at negative values when the system’s behavior remains constant over time. As the system experiences Hopf bifurcation, it attains a value of zero and again provides only negative values for a range of $\tau$. At the Hopf bifurcation point, the system changes from having a stable steady state to a stable periodic orbit, as observed in *y* and *z* variables in subfigures (c,d) of Fig. 3. The second-largest Lyapunov exponent $\lambda _2$ again takes the zero value while the system undergoes a period-doubling bifurcation. Furthermore, it acquires the value of zero during the appearance of a new stable periodic solution with twice the period of the original periodic attractor. In this way, the second-largest Lyapunov exponent helps to validate the points where period-doubling bifurcation occurs. It assumes a value of zero when $\tau$ is larger and close to 12, as the system experiences a series of period-doubling bifurcations. The third Lyapunov exponent $\lambda _3$ shown in light pink dotted line in Fig. 3a always remains negative throughout the interval of investigation. All these Lyapunov exponents help characterize the system’s dynamics and provide a better understanding of its sensitivity to initial conditions and predictability.

In spite of obtaining such captivating dynamics, we can not infer a few of these results from the biological viewpoint. The dynamics must be bounded within [0, 1]; otherwise when the total population exceeds the maximum value 1, we obtain an overcrowded solution. We plot a dash-dotted horizontal line in subfigures (a) and (c) of Fig. 3, below which the dynamics remain bounded within [0, 1]. Within the feasible range, we observe steady states as well as periodic oscillations in this figure. Apart from this dynamical behavior, our system (6) may depict quasiperiodic oscillation too, under a suitable choice of parameter values and initial conditions. The temptation parameter always plays a crucial role in determining the fate of defectors. If it is very large, defectors gain massive benefits that are too large to overcome. Under that circumstances, defectors are the unbeatable winners. If the temptation parameter is moderate, one can think about any approach to overcome the natural selfish instinct. To understand the effect of the temptation parameter in our study, we keep fixed all the parameter values and initial conditions as in Fig. 3 and choose three different pairs of values of $(\beta ,\tau )$. The dynamics offer a plethora of new behavior depending on these choices. We present all these observations in Fig. 4. Clearly, the temptation to defect can not help cooperators survive and evolve. Thus, we are never able to observe the survival of cooperators in Fig. 4 for the chosen parameter values and initial conditions. The cooperators’ fraction *x* always remains at zero. However, the dynamics of *y* and *z* again offer a wide variety. The chaotic dynamics observed in Fig. 3 is an overcrowded solution as $x+y+z>1$; hence we can not offer any biological interpretations for such an attractor. The upper panel of Fig. 4 shows a chaotic oscillation in *y* and *z* for $\beta = 1.93$ and $\tau = 11.64$; where $x+y+z \in [0,1]$. We plot the power spectrum corresponding to this temporal dynamics, which exhibits a broad range of frequencies in their power spectra, with no single dominant frequency. To avoid repetition, we chose not to display the Lyapunov exponent plot in this instance, but we have confirmed the validity of our results through its inclusion. Here for this set of parameter values, there is no ultimate winner among punishers and defectors, as they dominate one another in an unordered way, as revealed in subfigure (d). The chaotic oscillation never allows strict dominance over one another. Nevertheless, this scenario may alter for a different choice of $(\beta ,\tau )$, and one may dominate another in the long run. We choose $\beta =1.64$ and $\tau =10.73$ for the middle panel of this figure. The change in two parameter values does not alter the fortune of the cooperators, and they remain extinct in the long run, as shown in subfigure (f). However, maximum values of punishers’ density can dominate the defectors’ density for this set of parameter values (See subfigures (g–i)). We identify the oscillations that portray two-periodic dynamics. We plot the power spectrum of the time series in subfigure (j) and find these dominant frequencies correspond to the frequencies at which the signal repeats itself. All these subfigures confirm that the system (6) spends some time oscillating with one amplitude or frequency, then switching to the other amplitude or frequency, and continue oscillating in this way for these parameter values and initial conditions. The phase portrait in the *yz* plane in subfigure (i) of Fig. 4 also confirms this behavior.

The dynamical behavior is much more complex in the bottom panel of Fig. 4 where we choose $\beta =1.9$ and $\tau =10.5$. Cooperators are still unable to overcome the fierce struggle for resources and end up saturating in zero density, as shown in Fig. 4k. Punishers and defectors keep oscillating at two or more incommensurate frequencies, meaning that their ratio is irrational. The oscillation frequencies of these two populations exhibit a pattern that repeats itself over time, but the repetition is not strictly periodic (See subfigures (l–n)). The power spectrum of this attractor in of Fig. 4o confirms the appearance of a broad band of frequency components rather than distinct peaks at specific frequencies. And the width of these bands indicates the degree of incommensurability between the frequencies. This non-periodic and non-chaotic temporal behavior is a subject of deeper investigation and can provide insights into the behavior of complex systems. We would like to investigate the underlying mechanisms driving the system’s quasiperiodic dynamics and identify the key factors influencing its behavior in the future.

Figure 4 highlights the importance of these two parameters $\beta$ and $\tau$. In the following subsection, we will investigate the interplay of these two parameters in much more detail.

### Investigating the complex relationship between the temptation parameter and delay parameter

**Figure 5:**
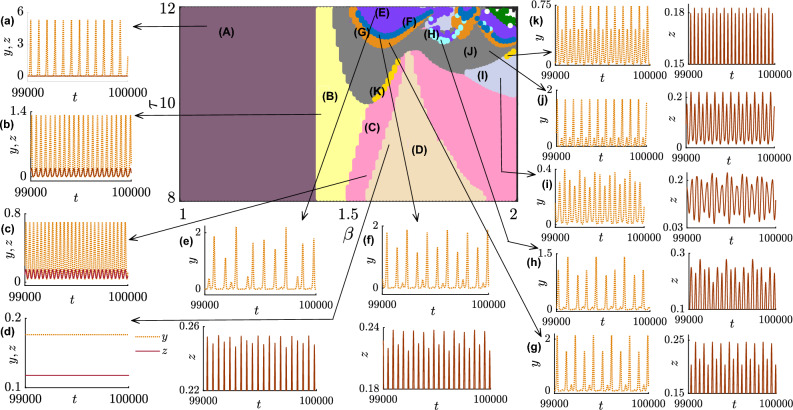
Two dimensional parameter space due to the simultaneous interplay of the temptation parameter $\beta$ and the delay parameter $\tau$: The $\beta -\tau$ parameter space unveils various temporal dynamics of different population variables in the region $\beta \in [1, 2]$ and $\tau \in [8, 12]$. The other parameter values are fixed at $\xi$ = 1.20, $\delta$ = 0.30, $\sigma _{1}$ = 1.35, $\sigma _{2}$ = 1.50, and $\sigma _{3}$ = 1.35, the initial conditions are set to be (0.1, 0.2, 0.5) and (0.3, 0.3, 0.3) for the non-delayed and delayed variables, respectively. Due to this particular choice of the parameter values both the cooperators and the defectors receive equal benefit from the free space (i.e., $\sigma _1 = \sigma _3$), however we end up with a society free from cooperators in the parameter region under study because of the initial advantage to the defectors over the cooperators. Time series of the punishers and defectors corresponding to different colored regions in the parameter space are shown, cooperators are not shown since they remain extinct throughout the space. The mauve region (A) on the left corresponds to a community only consisting of punishers that oscillate periodically. Time series in order to show oscillations in the player frequencies for this region are depicted by sub-figure (**a**). Periodic coexistence of both the punishers and defectors are observed in both the regions marked with lime yellow (B) and light pink (C) , but the region (B) corresponds to the overcrowded solution as $y + z > 1$, and the region (C) corresponds to the bounded solution. Sub-figure (**c**) highlights the periodic oscillatory time series data depicted by the of the region (C), whereas sub-figure (**b**) showcases the overcrowded scenario, marked by (B) region. Steady state coexistence of both punishers and defectors is noticed in the beige region (D) . The steady state time series data is marked in the sub-figure (**d**). Both the deep grey (J) and narrow yellow regions (K) correspond to period-2 oscillation in both the variables, however the deep grey region (J) indicates the overcrowded solution. Time series depictions are identified by sub-figures’ set (**k**), and (**j**) for the crowded and overcrowded two-periodic oscillatory states respectively. For better visibility, we do mark separate diagrams to portray two different oscillations of the player populations. Period-4 and period-8 oscillations emerge in the orange (G) and deep blue regions (F) , respectively, however again they correspond to overcrowded solution. We represent the population dynamics in order to showcase the four and eight periodic oscillations among the punishers and defectors by sub-figures’ set (**g**), and (**f**) respectively. Chaotic behavior is identified in the violet area (E) where the dynamics may or may not be bounded within 1. Chaotic behaviors among the two strategies are being shown in the set of sub-figures (**e**). Cyan (H) and light grey (I) colored regions exhibit period-6 and quasi periodic solutions in both the *y* and *z* variables, respectively. Period 6 oscillations are being shown in the set of sub-figures (**h**), whereas time series showing quasi-periodic oscillations are described in the sub-figures’ set (**i**). Deep green region corresponds to the extinction equilibrium (time series not shown). In the white region either the solution becomes unbounded or the sole existence of defectors is noticed.

Our proposed eco-evolutionary delay model explores various scenarios and player population diversities in society by changing the parameters in the model. The time delay parameter, $\tau$, is critical in altering the population dynamics, while the payoff parameters determine the viability of competing strategies in society. We analyze the survivability of different strategies and their dynamical behaviors by simultaneously varying two parameters: the temptation payoff parameter, $\beta$, and the time delay parameter, $\tau$, in a two-dimensional parameter space. We investigate the behavior of our model (6) by varying $\beta \in (1,2]$ and $\tau \in [8,12]$ with fixed step length 0.01, while keeping other parameters fixed at $\xi = 1.20$, $\delta =0.30$, $\sigma _{1}=1.35$, $\sigma _{2}=1.50$, and $\sigma _{3}=1.35$. The initial fractions for the non-delayed state variables are (0.1, 0.2, 0.5), while the initial fractions of the delayed variables are (0.3, 0.3, 0.3). This two-dimensional parameter space in Fig. 5 provides plenty of information about our proposed delayed system (6). In the classical prisoner’s dilemma game, defectors have a primary advantage over cooperators. We consider the chosen advantages from the free space as attributes to be equal for both the cooperator and defector populations ($\sigma _{1}=\sigma _{3}=1.35$). Moreover, the initial fraction of the population of cooperators is much lesser than others. Consequently, we end up with a society free from cooperators for any choice of temptation and delay parameters.

Since punishers receive significantly higher benefits ($\sigma _{2}=1.50$) from the free space compared to others, only the punishers are observed to survive periodically (indicated by mauve region (A) and by the time series presentation in sub-figure (a) in Fig. 5) while cooperators and defectors can not compete. In this regard, the fraction of the punisher population exceeds the threshold 1. When we vary the time delay by keeping the value of $\beta$ fixed, we observe an increase in the amplitude of oscillation but no change in the behavior of the competing strategies. However, an increment of $\beta$ always helps the defectors back in the contest. We locate a small area highlighted by lime yellow (B) in Fig. 5 where defectors can coexist with the punishers. Beyond $\beta =1.4$, the punishers and defectors begin to dominate one another depending on the time window and yield a periodic attractor here. Sub-figure (b) of Fig. 5 represents the periodic overcrowded oscillations among punishers and defectors. This periodic oscillation occurs due to the Hopf bifurcation, and the value of $\tau$, where the Hopf bifurcation occurs, gradually decreases as the value of $\beta$ increases. Notably, Ref.^13^ also found this critical value of $\beta =1.40$, beyond which defectors are very hard to defeat. As we further increase the temptation to defect, we observe an attractor with period-2 emerges at $\beta =1.46$ (See the set of sub-figures (j) along with the deep grey area (J) of Fig. 5). We further add the temporal behavior of different regions of the parameter space in Fig. 5. We observe the $z_{max}$ increases as $\beta$ increases. In other words, the progressive growth of $\beta$ facilitates the growth of the defector populations. However, the solution remains overcrowded as $y+z$ exceeds the upper bound of unity.

Noticeably, we are able to detect a fair portion, shown by light pink (C) in Fig. 5, where the overall population remains bounded within [0, 1]. We mark this particular kind of oscillations in sub-figure (c), and and in Fig. 5, pink region (C) is shown. The punishers and defectors oscillate periodically, allowing each to dominate the other depending on the time window. Even we are able to detect a very narrow yellow region (K) (See the sub-figures’ set (k) in Fig. 5) in the parameter space, where the dynamics of *y* and *z* variables exhibit periodic oscillations with period-2. In this region the overall population density $x+y+z$ remains within 1. Note that although society lacks the cooperators, punishers are also special kind of cooperators with some additional power to punish the defectors using their own resources. The absence of cooperators in the community is thus somehow controlled. More importantly, neither the punishers nor the defectors emerge as a dominant winner in this parameter space. Either of them can enjoy being dominant for a time window. However, their fate alters, and the other strategy dominates society for a different time course. Increasing the delay parameter $\tau$ leads to an amplification of oscillation amplitudes, which undergo period-doubling bifurcation as explained earlier. This results in oscillations with a period of 4 (timeseries for this oscillations are marked in sub-figure (g)), as highlighted in the orange section (G) of Fig. 5. Further increment of $\tau$ results in the appearance of eight periodic dynamics (presented in the set of sub-figures (f)) in the deep blue region (F) of Fig. 5, where the proportion of punishers and defectors oscillate. However, these orange and deep blue regions occur only in overcrowded solutions, where the sum of $x+y+z$ exceeds 1. As we continue to increase the value of $\tau$, our model (6) may exhibit chaotic behavior, as indicated by the violet area (E) in the $\beta -\tau$ parameter space. The chaotic phenomenon among the two strategies are portrayed by the set of sub-figures (e) in Fig. 5. In this region, the sum of $x+y+z$ may or may not be bounded within 1. Our findings are supported by power spectrum analysis, but these results are not presented here to avoid redundancy.

Figure 5 also displays a beige colored region (D) where the system (6) converges to a steady state with no cooperators. The nature in the player population frequencies for the steady states are shown in the sub-figure (d) in Fig. 5. Furthermore, the system (6) may converge to the extinction equilibrium (0, 0, 0), shown by deep green in Fig. 5, resulting in a population-free state (time series not shown). In the next section, we will perform an analytical calculation for each of these steady states. The white region in the parameter space is identified as a region where the solution may become unbounded or converge to the steady state $\bigg (0,0,1-\dfrac{\xi }{\sigma _{3}}\bigg )$. This disappearance of attractors is similar to what is discussed in Ref.^46^. In the future, we aim to investigate the underlying cause of this disappearance of attractors in our model. It is worth noting from an ecological perspective that the steady-state solution is now bounded within the range of [0, 1]. Interestingly, the cooperator’s survivability cannot be facilitated by either the temptation or delay parameter in the entire domain of analysis. Increasing the value of $\beta$ only helps to promote the density of defectors depending on the initial conditions and other parameter values. The delay parameter leads to the emergence of periodic behavior, allowing the populations to dominate each other regularly. However, periodic and chaotic attractors are not the only possible outcomes that can be obtained by varying the parameters. In Fig. 5, we identify a light purple region where the density of punishers and defectors display quasi periodic oscillations that remain bounded within the range of [0, 1]. We additionally identify a regime, shown by cyan (H) in Fig. 5, where both *y* and *z* variables oscillate with six periodic dynamics . We present the set of sub-figures (h) to describe the six-periodic oscillation among the competing population traits. Moreover, before the note of a period doubling phenomenon from periodic oscillation to bi-periodic oscillation, we also observe quasi-periodicity among punisher and defector traits, which has been marked by light grey region (I) in Fig. 5. we present the quasi-periodic oscillations in the sub-figures’ set (i) of Fig. 5.

In the upcoming subsection, we will shed light on the impact of additional system parameters on our model. Specifically, we will investigate the effects by varying two different parameters while keeping the others fixed. By doing so, we aim to uncover valuable insights into the influence of these specific parameters on our model’s behavior.

### System parameters and eco-evolutionary dynamics: unveiling the hidden connections

**Figure 6:**
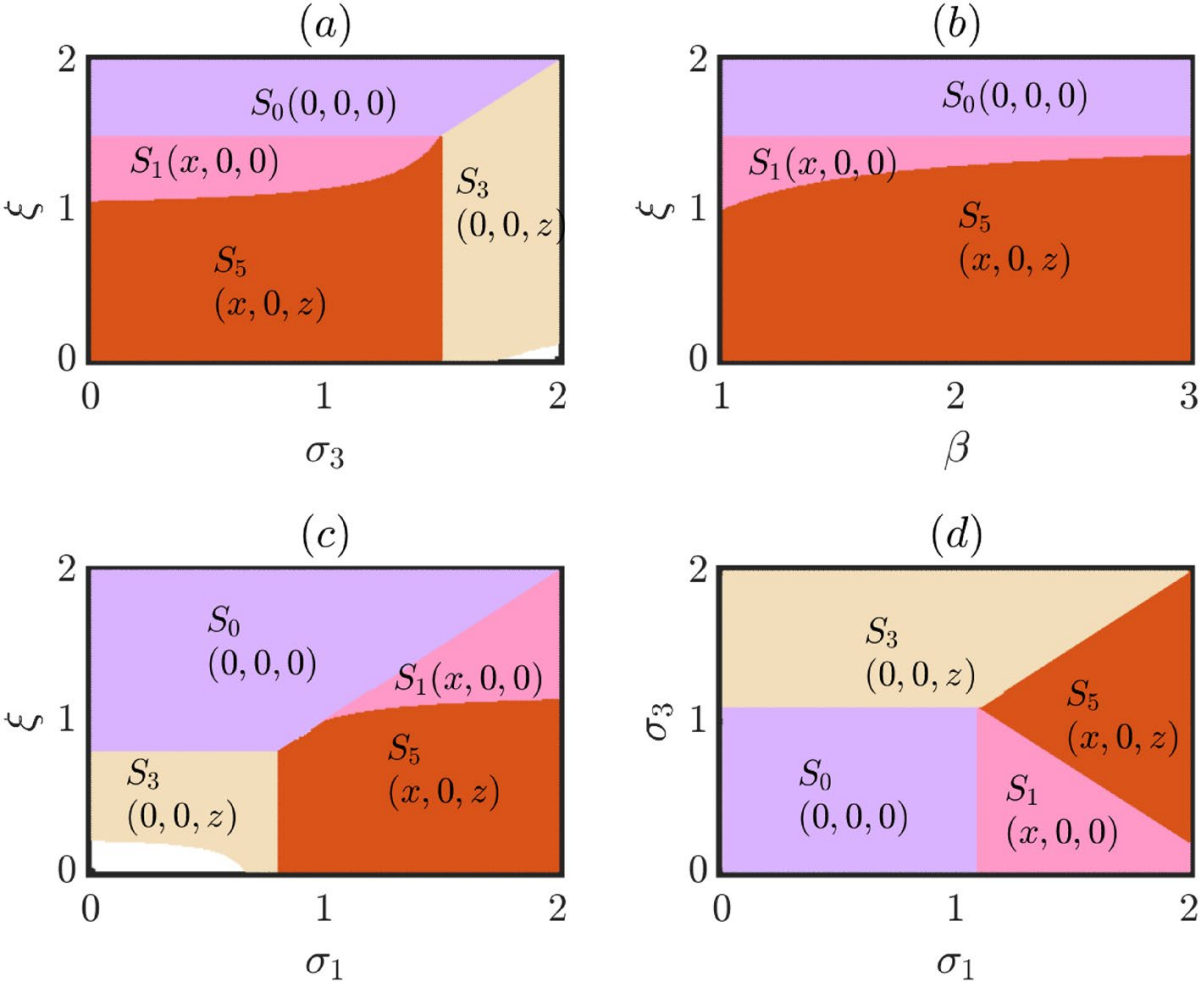
Exploring the role of system parameters in eco-evolutionary dynamics: Subfigure (**a**) shows that as the mortality rate increases, all strategies face extinction unless free space benefits defectors substantially, leading to their dominance. A significant portion of the parameter space allows for the coexistence of cooperators and defectors, while another portion enables the survival of cooperators alone. Similarly, in subfigure (**b**), escalating mortality rates result in the extinction of all strategies, except in a region where only cooperators survive. However, this region diminishes with higher temptation. This parameter space exhibits the coexistence of cooperators and defectors, also. In subfigure (**c**), increasing benefits for cooperators, while keeping other parameters fixed, leads to a surge in their density. Higher mortality rates pose a challenge, but cooperators can persist due to their enhanced benefits. A small region, shown in white, exhibits unbounded dynamics in subfigures (**a**) and (**c**). Subfigure (**d**) illustrates the delicate balance between benefits for defectors and cooperators. When defectors have a greater advantage and exceed the mortality rate, they become the sole survivors. Cooperators can coexist with defectors or thrive as the sole strategy when their benefits surpass both the mortality rate and defectors’ benefits. Punishers are absent in the whole figure due to chosen parameters and initial conditions, highlighting the intriguing dynamics. Parameter values: $\sigma _1=1.2$, $\sigma _2=1.5$, $\sigma _3=1.4$, $\xi =1.1$, $\beta =1.5$, $\delta =0.5$, and $\tau =0.2$, unless those parameters are varied. Initial conditions for delayed variables: (0.25, 0.25, 0.25), and for non-delayed variables: (0.3, 0.3, 0.3).

In the context of our study, it becomes evident that when free space presents a more favorable opportunity for defectors, while keeping other parameter values constant, their survival becomes highly likely. In such scenarios, if the benefit induced by free space towards defectors, denoted as $\sigma _3$, reaches a sufficiently large value, the other strategies face intense competition and may eventually disappear from the societal dynamics. This observation is depicted in Fig. 6a, where we observe that an increase in the mortality rate $\xi$ leads to an equilibrium of extinction, rendering no viable survivors. Conversely, when the mortality rate is moderate, the system exhibits the potential to sustain a society solely composed of cooperators, contingent upon other parameter considerations. Further reducing the mortality rate enables the coexistence of cooperators and defectors. However, an amplification of the free space-induced benefit toward defectors favors their dominance, ultimately eliminating cooperators from the competition. Consequently, under such circumstances, defectors emerge as the sole surviving strategy.

An analogous observation is evident when examining Fig. 6b, which further accentuates this phenomenon of interest. Once again, we observe that as mortality rate escalates, no strategies are able to evolve, ultimately leading to their eventual extinction over extended time period. However, within the expansive $\beta -\xi$ parameter space, a distinct region emerges where the survival of solely cooperators becomes feasible. It is important to note, however, that this region diminishes in size as the temptation parameter $\beta$ increases. This correlation arises from the fact that defectors receive an augmented advantage with a higher $\beta$, thereby reducing the space where the sole coexistence of cooperators can only occur. In fact, it is within the moderate range of mortality rate that the majority of the parameter space allows for the coexistence of defectors and cooperators.

In a similar vein, if we delve into the intricate interplay of free space-induced benefits towards cooperators, specifically $\sigma _1$, while keeping $\sigma _3$ and other parameters fixed, we anticipate a noticeable surge in the density of cooperators, denoted by *x*, with each incremental value of $\sigma _1$. The exquisite Fig. 6c illuminates the captivating dynamics that unfold within the system. For lower values of $\sigma _1$, the system may initially lack any cooperators. However, as we venture beyond a critical threshold of $\sigma _1$, contingent upon the values of other parameters, cooperators reveal their resilience, persisting in the system over extended temporal horizons. While a higher mortality rate poses a formidable barrier to the survival of any individual, the enhanced free space-induced benefits bestowed upon cooperators hold the potential for their sustenance, even under such adversities. Moreover, within the expanse of the $\sigma _1-\xi$ parameter space, a significant portion emerges where only defectors can endure due to the relatively diminished contribution of free space towards cooperators. Nevertheless, as the value of $\sigma _1$ rises, cooperators resurge, reentering the competitive landscape and forging a coexistence alongside defectors. However, it is crucial to acknowledge the existence of a small region within this parameter space where the dynamics become unbounded over sufficiently long time frames. The intriguing phenomenon of unboundedness manifests itself in a similar fashion within Fig. 6a as well. In the near future, we aspire to delve deeper into this enthralling phenomenon, unveiling the precise mechanisms underlying this unboundedness.

Figure 6d vividly illustrates the intricate interplay between the parameters $\sigma _1$ and $\sigma _3$. Notably, when both of these parameters are lesser than the mortality rate $\xi =1.10$, the system reaches an equilibrium of extinction, effectively stabilizing the dynamics in an unwanted scenario. When free space provides defectors with a more favorable opportunity compared to cooperators (indicated by $\sigma _3 > \sigma _1$) and $\sigma _3$ surpasses the mortality rate $\xi$, defectors gain substantial advantages, thereby emerging as the sole surviving strategy within that specific parameter space. On the other hand, if $\sigma _1$ exceeds both $\xi$ and $\sigma _3$, two distinct scenarios unfold, favoring the survivability of cooperators. In one scenario, the parameter choices allow for the coexistence of cooperators and defectors, contingent upon the specific values of $\sigma _3$. In the second scenario, cooperators thrive as the sole surviving strategy in the societal dynamics, while all others face extinction. It is worth noting that, due to our chosen parameter values and initial conditions, Fig. 6 remains devoid of any punishers, underscoring the fascinating dynamics at play within the system.

**Figure 7:**
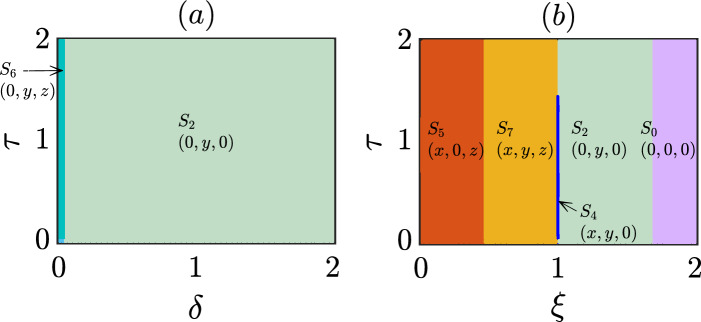
Unveiling the interplay of delay, mortality rate, and punishment in curbing selfish behavior of defectors: (**a**) Corresponds to the exploration of the response to simultaneous variations of $\delta$ and $\tau$, uncovering a pattern of strategy survival shaped by fine imposition. Coexistence between punishers and defectors prevails at minimal punishment while escalating punishment leads to the dominance of punishers and the extinction of defectors. These outcomes depend on our specific parameter values, emphasizing the delicate balance between temptation, hierarchy, and strategy survival. (**b**) Unveils the intricate behavior of our model when varying $\xi$ and $\tau$, shedding light on the interplay between mortality rate and strategy dynamics. High mortality erodes all strategies, but reducing $\xi$ allows for a society of sole punishers. Coexistence between cooperators and punishers emerges in a very narrow parameter region, free from defectors. The influence of the delay parameter $\tau$ is limited within the investigated range, except for a thin white portion with unbounded dynamics. Decreasing $\xi$ further leads to the coexistence of all strategies, fostering biodiversity, and eventually, to a society of cooperators and defectors. Parameter values: $\sigma _1=1.2$, $\sigma _2=1.5$, $\sigma _3=1.4$, $\xi =1.1$, $\beta =1.5$, $\delta =0.5$, and $\tau =0.2$, unless those parameters are varied. Initial conditions for delayed variables: (0.25, 0.25, 0.25), and for non-delayed variables: (0.3, 0.3, 0.3).

Intriguing insights into the behavior of our proposed model await us as we explore the captivating Fig. 7. Remarkably, we discover that the dynamics remain almost unaffected by variations in the delay parameter, despite we have found a profound effect of $\tau$ in our study through several earlier discussions (e.g., see Fig. 5). Within Fig. 7a, we embark on a journey to explore the system’s response when simultaneously varying two parameters, namely $\delta$ and $\tau$, while maintaining fixed values for other parameters at $\sigma _1=1.2$, $\sigma _2=1.5$, $\sigma _3=1.4$, $\xi =1.1$, and $\beta =1.5$. A discernible pattern emerges from our investigation, shedding light on the interplay between fine imposition and the survival prospects of different strategies. At minimal fine levels, coexistence between punishers and defectors prevails within the societal landscape. However, as we escalate the fine magnitude, defectors too succumb to their inability to thrive, leaving behind a society solely populated by punishers. It is worth noting that these outcomes may exhibit variations when other parameter values are chosen differently. Our selection of parameter values deliberately maintains a moderate temptation to defect ($\beta =1.5$) while endowing punishers with a more advantageous position in the free space hierarchy ($\sigma _2>\sigma _3>\sigma _1$). Consequently, cooperators, receiving the least support from free space, struggle to sustain themselves and ultimately face extinction in the long run. Furthermore, this subfigure, Fig. 7a, stands devoid of cooperators, underscoring the inhospitable environment created by the combined influence of fine imposition and delay on their survival prospects. The aforementioned exploration illuminates the intricate dynamics at play, deepening our understanding of the complex interactions within the proposed model.

The captivating Fig. 7b unravels the intricate behavior of our model as we simultaneously vary the parameters $\xi$ and $\tau$, while holding the remaining parameters constant at the same values as in Fig. 7a with $\delta =0.5$. Within this visual exploration, we witness the system’s remarkable response to these parameter variations, elucidating the delicate balance that governs the survival and coexistence of different strategies. As the mortality rate $\xi$ escalates towards high values, approaching 2, a disheartening scenario unfolds where no strategies manage to endure in the long run. This highlights the adverse consequences of an excessively high mortality rate within the system. However, by reducing the strength of the mortality rate $\xi$, a fascinating transformation occurs, leading to the emergence of a society comprised solely of punishers. This finding aligns with the insights gleaned from Fig. 7a, highlighting the intricate relationship between mortality rate and strategy dynamics. It is noteworthy to mention that in our proposed model, punishers themselves embody a distinct form of cooperation. Intriguingly, within the two-dimensional parameter space with $\xi =1.0$, we discover a region where cooperators and punishers coexist harmoniously, while defectors remain absent from the societal landscape. This coexistence phenomenon further emphasizes the complex interplay between the investigated parameters. However, as we examine the intricacies portrayed in Fig. 7b, we observe that the delay parameter $\tau$ exhibits limited influence on the emerging state within the investigated range. Notably, a thin white portion in the parameter space for $\xi =1.0$ unveils the emergence of unbounded dynamics, warranting further investigation into the precise mechanisms behind this intriguing phenomenon. By decreasing the value of the mortality rate $\xi$ even further, we uncover a substantial portion within the parameter space that facilitates the coexistence of all three strategies, fostering biodiversity within the system. As the mortality rate $\xi$ continues to decrease, we witness a captivating transformation where punishers struggle to survive, ultimately leading to a society where cooperators and defectors coexist in delicate harmony. This profound exploration offers invaluable insights into the complex dynamics of our model, unraveling the delicate relationships between mortality rate, delay parameter, and the survival prospects of different strategies.

Both Figs. 6 and 7 showcase numerical simulations based on a fixed initial condition. Specifically, the non-delayed variables are initialized with values of (0.3, 0.3, 0.3), while the delayed variables begin with values of (0.25, 0.25, 0.25). These initial conditions serve as the starting point for investigating the intriguing interplay of parameters in our proposed model and their influence on the emergence of eco-evolutionary dynamics. In the subsequent subsection, we will delve into the pivotal role of initial conditions in our model, analyzing how they shape and contribute to the complex dynamics observed within the system. By examining the impact of different initial conditions, we aim to gain a deeper understanding of the intricate relationships between system parameters, initial states, and the resulting eco-evolutionary dynamics.

### Uncovering multistability: insights into eco-evolutionary dynamics

**Figure 8:**
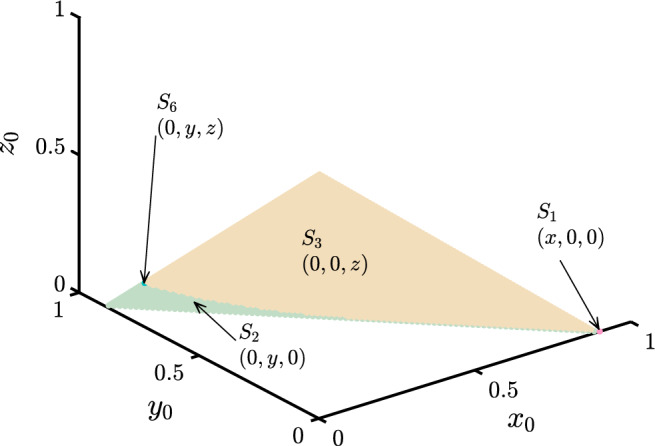
Exploring the impact of initial conditions on eco-evolutionary dynamics: Varying initial conditions within the constraint $x_0+y_0+z_0=0.9$, we uncover diverse steady states, highlighting the profound influence of initial conditions on emergent dynamics. This multistability provides valuable insights into system stability, robustness, and regime shifts. Notably, our findings reveal four distinct attractors representing different stable strategies, including coexistence of punishers and defectors. Depending on the chosen initial conditions, the system can exhibit stable states with cooperators, punishers, or defectors alone. These discoveries deepen our comprehension of the intricate dynamics and potential outcomes in our eco-evolutionary model, underscoring the critical interplay between initial conditions and steady states. Parameter values: $\sigma _1=1.2$, $\sigma _2=1.5$, $\sigma _3=1.4$, $\xi =1.1$, $\beta =1.5$, $\delta =0.5$, and $\tau =0.2$. Initial conditions for delayed variables: (0.25, 0.25, 0.25).

We maintain the parameter values at $\sigma _1=1.2$, $\sigma _2=1.5$, $\sigma _3=1.4$, $\xi =1.1$, $\beta =1.5$, $\delta =0.5$, and $\tau =0.2$, consistent with our earlier investigations in Figs. 6 and 7. However, in this subsection, we focus on the role of initial conditions in our model. To explore the range of possible outcomes, we vary the initial conditions $(x_0,y_0,z_0)$ while satisfying the constraint $x_0+y_0+z_0=0.9$. This constraint allows us to interpret the results from a biological perspective, as $x_0+y_0+z_0$ falls within the range of [0, 1]. Similar to our previous investigations, we set the initial condition for the non-delayed variables as (0.25, 0.25, 0.25). Remarkably, we observe that the system converges to different steady states depending on the chosen initial conditions. This finding further reinforces our earlier claim that the emergent dynamics critically depend on the initial conditions. The coexistence of multiple stable states in our model offers valuable insights into the system’s stability, robustness, and the possibility of regime shifts. In particular, in Fig. 8, we present the results that reveal the existence of four distinct attractors. These attractors represent different stable strategies that can persist over time. Under specific conditions, we can observe the coexistence of punishers and defectors within the system. Moreover, depending on the initial conditions, the system may exhibit stable states consisting solely of cooperators, punishers, or defectors. The identification of these multiple stable states sheds light on the complex dynamics and potential outcomes in our eco-evolutionary model. Understanding the interplay between initial conditions and the resulting steady states is crucial for comprehending the long-term behavior and potential shifts in ecological and evolutionary systems.

In the upcoming section, we derive the steady states and analytically determine the point of Hopf bifurcation in our model. Through rigorous analysis and numerical validation, we establish the accuracy of our findings.

## Analytical results

### Various steady states and their biological relevance

The proposed model (6) results in eight steady states. They are briefly discussed in the following,

- *The extinction state* $S_0$: The state of extinction, denoted as $S_0$, corresponds to the equilibrium point (0, 0, 0) in the dynamical system. At this state, each of the player populations eventually die out due to intense competition over long periods of time.
- *Punisher and Defector-free state* $S_1$: In this steady state, only individuals with cooperative strategy have the opportunity to survive, leading to the extinction of all other types of players in the game. The stationary point associated with this state is given by $\bigg (\dfrac{\sigma _{1}-\xi }{\sigma _{1}-1},0,0\bigg )$. Under these circumstances, cooperators have the ability to survive and ultimately dominate the entire game.
- *Cooperator and defector-free state* $S_2$: This state exclusively enables the survival of punishers, resulting in the eventual extinction of all other populations in the game. The stationary point associated with this state is $\bigg (0,\dfrac{\sigma _{2}-\xi }{\sigma _{2}-1},0\bigg )$. In this scenario, punishers gain the most significant advantage and can solely dominate the entire game. However, in an interactive context, if a society consists of exclusively punishers, they would act as cooperators because there would be no defectors to punish.
- *Cooperator and punisher-free state* $S_3$: This state allows only defectors to survive in a society, which supports the fundamental theory of the prisoner’s dilemma game, as defectors receive the greatest benefit compared to all other population strategies. The equilibrium point associated with this state is $\bigg (0,0,1-\dfrac{\xi }{\sigma _{3}}\bigg )$. In such scenario, where only one population strategy can survive in the long run, there will be no interactive action between different player populations with different strategies.
- *Defector-free state* $S_4$: In this steady state, cooperators and punishers get the chance to survive side by side, with no defectors in the interaction. The equilibrium point gets the form $(x_*,y_*,0)$, where, $x_*+y_*$ = $\dfrac{\sigma _{1}-\xi }{\sigma _{1}-1}$. Here, the defectors are unable to survive, whereas, the cooperators and the punishers jointly survive. Punishers also behave like cooperators in such state.
- *Punisher-free state* $S_5$: This state in the proposed model represents the basic scenario of the prisoner’s dilemma game. In the long run, only the cooperators and defectors interact with each other, with no punishers present in the society. In such a scenario, the chances of implementing cooperation in different ways are reduced because the defector is not constrained in dealing with cooperators in the absence of punishers. The stationary point can be expressed as $(x_*,0,z_*)$, where $x_*= \dfrac{\xi (\sigma _{1}-\sigma _{3})}{\beta \sigma _{1}-\sigma _{3}}$, and $z_*=1-x_*+\dfrac{\xi (1-\beta )}{\beta \sigma _{1}-\sigma _{3}}$.
- *Cooperator-free state* $S_6$: This steady state operates similarly to the previous state, $S_5$, where, in the long run of the dilemma, the punishers and the defectors can interact with each other. However, unlike state $S_5$, the defectors in this state face a slight constraint when interacting with the punishers. There would be a slight reduction in their payoffs when interacting with punishers than the cooperators. On the other hand, the punishers also behave like cooperators, but when interacting with the defectors, they face a loss of the fine $\delta$ to punish the defectors. The equilibrium point is given by $(0,y_*,z_*)$, where $y_*= \dfrac{\xi (\sigma _{2}-\sigma _{3})+\delta (\xi -\sigma _{3})}{(\beta \sigma _{2}-\sigma _{3})+\delta (\beta -\delta -\sigma _{2}-\sigma _{3})}$, and $z_*=1-y_*+\dfrac{\xi (1-\beta +2\delta )-\delta (\beta -\delta )}{(\beta \sigma _{2}-\sigma _{3})+\delta (\beta -\delta -\sigma _{2}-\sigma _{3})}$.
- *State of co-existence* $S_7$: This steady state allows all three types of player populations to coexist and interact with each other simultaneously. It is considered the most biologically significant among all eight states as no player population goes extinct in the long run. The equilibrium point for this state is $(x_*, y_*, z_*)$, where $x_* = -\gamma _1 - \alpha _1 - z_* + \delta _1$, $y_* = \alpha _1 + \gamma _1$, and $z_* = \dfrac{(\xi - 1)(\sigma _{2} - \sigma _{1})}{\sigma _{1} - \sigma _{2} - \delta + \sigma _{1}\delta }$. Here, $\gamma _1 = \dfrac{(\beta - 1)[\xi (\sigma _{1} - \sigma _{2} - \delta ) + \sigma _{1}\delta ]}{\sigma _{1} - \sigma _{2} - \delta + \sigma _{1}\delta }$, $\alpha _1 = \dfrac{(1-\xi )(\sigma _{1}-\sigma _{3})}{\sigma _{1}-\sigma _{2}-\delta +\sigma _{1}\delta }$, $\delta _1 = 1 + \dfrac{\delta (1-\xi )}{\sigma _{1}-\sigma _{2}-\delta +\sigma _{1}\delta }$, and $\sigma _{1}-\sigma _{2}-\delta +\sigma _{1}\delta \ne 0$.

### Tracking the point of occurrence of Hopf bifurcation

Assuming $(x_*, y_*, z_*)$ as the coordinates of a specific steady state, we explore the occurrence of the Hopf bifurcation by progressively increasing the time delay variable $\tau$ imposed on the system from this stable steady state. We refer to the value of $\tau$ at which the steady states begin to lose stability as the critical value of $\tau$ or $\tau _c$. The linearization form of the proposed system (6) is presented below.

$$\begin{aligned}{} & {} \dot{x}-[(1-\sigma _{1})xx_\tau + (1-\sigma _{1})xy_\tau -\sigma _{1}xz_\tau +(\sigma _{1}-\xi )x]=0,\\{} & {} \dot{y}-[(1-\sigma _{2})yx_\tau + (1-\sigma _{2})yy_\tau -(\delta +\sigma _{2})yz_\tau +(\sigma _{2}-\xi )y] =0, \\{} & {} \dot{z}-[(\beta -\sigma _{3})z x_\tau +(\beta -\delta -\sigma _{3})zy_\tau -\sigma _{3}zz_\tau +(\sigma _{3}-\xi )z] =0. \end{aligned}$$

By calculating the linearized version of the above system about the general steady state $(x_*,y_*,z_*)$, we obtain

7 $$\begin{aligned} \dot{x}=&\, {} \psi _{11}x+\psi _{12}x_\tau +\psi _{13}y_\tau +\psi _{14}z_\tau ,\nonumber \\ \dot{y}=& \,{} \psi _{21}y+\psi _{22}x_\tau +\psi _{23}y_\tau +\psi _{24}z_\tau ,\nonumber \\ \dot{z}=&\, {} \psi _{31}z+\psi _{32}x_\tau +\psi _{33}y_\tau +\psi _{34}z_\tau , \end{aligned}$$

where

8 $$\begin{aligned} \psi _{11}=&\, {} (1-\sigma _{1})(x_*+y_*)-\sigma _{1}z_*+(\sigma _{1}-\xi ),\nonumber \\ \psi _{12}=& {}\, (1-\sigma _{1})x_*=\psi _{13},\nonumber \\ \psi _{14}=&\, {} -\sigma _{1}x_*,\nonumber \\ \psi _{21}=& {}\, (1-\sigma _{2})(x_*+y_*)-(\delta +\sigma _{2})z_*+(\sigma _{2}-\xi ),\nonumber \\ \psi _{22}=&\, {} (1-\sigma _{2})y_*=\psi _{23},\nonumber \\ \psi _{24}=&\, {} -(\delta +\sigma _{2})y_*,\nonumber \\ \psi _{31}=&\, {} (\beta -\sigma _{3})x_*+(\beta -\delta -\sigma _{3})y_*-\sigma _{3}z_*+(\sigma _{3}-\xi ),\nonumber \\ \psi _{32}=& \,{} (\beta -\sigma _{3})z_*,\nonumber \\ \psi _{33}=& {} \,(\beta -\delta -\sigma _{3})z_*,\nonumber \\ \psi _{34}=&\, {} -\sigma _{3}z_*. \end{aligned}$$

The characteristic equations of the linearized system (7) are obtained as $\dot{S}=JX$, where

$$\begin{aligned} \dot{S}=\begin{bmatrix} sx(s) \\ sy(s) \\ sz(s) \end{bmatrix}, J=\begin{bmatrix} \psi _{11}+\psi _{12}e^{-s\tau } &{} \psi _{13}e^{-s\tau } &{} \psi _{14}e^{-s\tau } \\ \psi _{22}e^{-s\tau } &{} \psi _{21}+\psi _{23}e^{-s\tau } &{} \psi _{24}e^{-s\tau } \\ \psi _{32}e^{-s\tau } &{} \psi _{33}e^{-s\tau } &{} \psi _{31}+\psi _{34}e^{-s\tau } \\ \end{bmatrix}, \text { and } X=\begin{bmatrix} x(s) \\ y(s) \\ z(s) \end{bmatrix}. \end{aligned}$$

In this case, *J* represents the Jacobian of the linearized system of equations. To obtain its characteristic equation, we set $det(J-\lambda I)=0$, where $\lambda$ is an eigenvalue of the Jacobian matrix *J*. From the resulting eigenvalues, we can deduce the critical value of $\tau$. Therefore, we have the following transcendental equation from $det(J-\lambda I)=0$:

9 $$\begin{aligned} \lambda ^{3}+\nu _1 \lambda ^{2}+\nu _2 \lambda +(\nu _3 \lambda ^2+\nu _4 \lambda +\nu _5)e^{-\lambda \tau }+(\nu _6\lambda +\nu _7)e^{-2\lambda \tau }+\nu _8 e^{-3\lambda \tau }+\nu _9=0, \end{aligned}$$

where

10 $$\begin{aligned} \nu _1=& {} \, -(\psi _{11}+\psi _{21}+\psi _{31}),\nonumber \\ \nu _2=& {}\, \psi _{11}\psi _{21}+\psi _{11}\psi _{31}+\psi _{21}\psi _{31},\nonumber \\ \nu _3=& {} \, -(\psi _{12}+\psi _{23}+\psi _{34}),\nonumber \\ \nu _4=& {}\, \psi _{11} \psi _{23}+\psi _{11}\psi _{34}+\psi _{12}\psi _{21}+\psi _{12}\psi _{31}+\psi _{21}\psi _{34}+\psi _{23}\psi _{31},\nonumber \\ \nu _5=& {}\, -(\psi _{11}\psi _{21}\psi _{34}+\psi _{11}\psi _{23}\psi _{31}+\psi _{12}\psi _{21}\psi _{31}),\nonumber \\ \nu _6=& {}\, -(\psi _{24}\psi _{33}-\psi _{12}\psi _{23}-\psi _{12}\psi _{34}-\psi _{23}\psi _{34}+\psi _{13}\psi _{22}+\psi _{14}\psi _{32}),\nonumber \\ \nu _7=& {}\, -(\psi _{11}\psi _{23}\psi _{34}-\psi _{11}\psi _{24}\psi _{33}+\psi _{12}\psi _{21}\psi _{34}+\psi _{12}\psi _{23}\psi _{31}-\psi _{13}\psi _{22}\psi _{31}-\psi _{14}\psi _{21}\psi _{32}),\nonumber \\ \nu _8=& {}\, -(\psi _{12}\psi _{23}\psi _{34}-\psi _{12}\psi _{24}\psi _{33}-\psi _{13}\psi _{22}\psi _{34}+\psi _{13}\psi _{24}\psi _{32}+\psi _{14}\psi _{22}\psi _{33}-\psi _{14}\psi _{32}\psi _{23}),\nonumber \\ \nu _9=& {}\, - \psi _{11}\psi _{21}\psi _{31}. \end{aligned}$$

To simplify the transcendental equation, we make the following substitution: $\lambda (\tau )=a(\tau )+ib(\tau )$ and $\lambda (\tau _c)=a(\tau _c)+ib(\tau _c)$. We then separate the real and imaginary parts of the equation by using the transformation $e^{a\pm ib}=e^{a}(cos(b)\pm isin(b))$ on Eq. (9). This yields

11 $$\begin{aligned}{} & {} a^3-3ab^2+\nu _1(a^2-b^2)+a\nu _2+(\nu _3(a^2-b^2)+a\nu _4 +\nu _5)e^{-a\tau }\cos (a\tau )+\nonumber \\{} & {} \quad \{2\nu _3ab+\nu _4b\}e^{-a\tau }\sin (b\tau )+(a\nu _6+\nu _7)e^{-2a\tau }\cos (2b\tau )+\nonumber \\{} & {} \quad b\nu _6e^{-2a\tau }\sin (2b\tau )+\nu _8e^{-3a\tau }\cos (3b\tau )+\nu _9=0, \text {and}, \nonumber \\{} & {} \quad 3a^2b-b^3+2\nu _1ab+b\nu _2-a\sin (b\tau )-\{\nu _3(a^2-b^2)+a\nu _4+\nu _5\}e^{-a\tau } \sin (b\tau )+\{2\nu _3ab+b\nu _4\}\nonumber \\{} & {} \quad e^{-a\tau }\cos (b\tau )-(a\nu _6+\nu _7)e^{-2a\tau }\sin (2b\tau )+b\nu _6e^{-2a\tau } \cos (2b\tau )-\nu _8e^{-3a\tau }\sin (3b\tau )=0. \end{aligned}$$

Using the relation in Eq. (11), we can determine the value of the delay time variable $\tau _c$ at which the Hopf bifurcation occurs. At this value of $\tau$, the eigenvalues of the Jacobian become purely imaginary. Therefore, we set $a(\tau _c)=0$. This provides the following form of Eq. (11):

12 $$\begin{aligned}{} & {} (b^{*2}\nu _3-\nu _5)\cos (b^*\tau _c)-b^*\nu _4\sin (b^*\tau _c)=\nu _7\cos (2b^*\tau _c)+\nu _6b^*\sin (2b^*\tau _c)\nonumber \\{} & {} \quad +\nu _8\cos (3b^*\tau _c)+\nu _9-b^{*2}\nu _1, \text {and}, \nonumber \\{} & {} \quad b^*\nu _4\cos (b^*\tau _c)+(b^{*2}\nu _3-\nu _5)\sin (b^*\tau _c)=\nu _7\sin (2b^*\tau _c)-b^*\nu _6\cos (2b^*\tau _c)+\nonumber \\{} & {} \quad \nu _8\sin (3b^*\tau _c)+b^{*3}-b^{*}\nu _2. \end{aligned}$$

Let us consider that

$$\begin{aligned} A=& \, {} (b^{*2}\nu _3-\nu _5)\cos (b^*\tau _c)-b^*\nu _4\sin (b^*\tau _c),\\ B=& \,{} \nu _7\cos (2b^*\tau _c)+\nu _6b^*\sin (2b^*\tau _c)+\nu _8\cos (3b^*\tau _c),\\ C=& \, {} b^*\nu _4\cos (b^*\tau _c)+(b^{*2}\nu _3-\nu _5)\sin (b^*\tau _c), \text {and}\\ D=& {} \, \nu _7\sin (2b^*\tau _c)-b^*\nu _6\cos (2b^*\tau _c)+\nu _8\sin (3b^*\tau _c). \end{aligned}$$

Consequently, we find the following relations,

$$\begin{aligned} A-B&=\nu _9-b^{*2}\nu _1,\\ C-D&=b^{*3}-b^*\nu _2,\\ \end{aligned}$$

Using these two relations, we can easily conclude

13 $$\begin{aligned} (A^2+C^2)+(B^2+D^2)-2(AB+CD)=b^{*6}+(\nu _1^2-2\nu _2)b^{*4}-(2\nu _1\nu _9-\nu _2^2)b^{*2}+\nu _9^2 
\end{aligned}$$

Since the left-hand side of Eq. (13) includes the terms $(A-B)^2$ and $(C-D)^2$, and considering the assumptions we have made about these values, we can substitute them and rewrite Eq. (13) as follows:

14 $$\begin{aligned}{} & {} b^{*4}\nu _3^2+\nu _5^2-2\nu _3\nu _5b^{*2}+b^{*2}\nu _4^2+\nu _7^2+b^{*2}\nu _6^2+\nu _8^2 +2\nu _7\nu _8\cos (b^*\tau _c)-2\nu _6\nu _8b^*\sin (b^*\tau _c)\nonumber \\{} & {} \quad -2(\nu _3\nu _7b^{*2}\cos (2b^*\tau _c)+\nu _3\nu _6b^{*3}\sin (b^*\tau _c)+\nu _3\nu _8b^{*2} \cos (2b^*\tau _c)-\nu _5\nu _7\cos (b^*\tau _c)-\nu _5\nu _8b^*\sin (b^*\tau _c)\nonumber \\{} & {} \quad -\nu _5\nu _8\cos (2b^*\tau _c)+\nu _4\nu _7b^*\sin (b^*\tau _c)-\nu _4\nu _6b^{*2}cos(b^*\tau _c) +\nu _4\nu _8b^*\sin (2b^*\tau _c))=\nonumber \\{} & {} \quad b^{*6}+(\nu _1^2-2\nu _2)b^{*4}-(2\nu _1\nu _9-\nu _2^2)b^{*2}+\nu _9^2. \end{aligned}$$

After performing extensive calculations, we can derive the following relationship by multiplying $\cos (2b^*\tau _c)$ with the first equation of system (12) and $\sin (2b^*\tau _c)$ with the second equation of the same system.

15 $$\begin{aligned} \sin {(b^* \tau _c)}= \dfrac{c+d\cos {(b^*\tau _c)}+e\cos {(2b^*\tau _c)}-a\cos {(b^*\tau _c)}}{b-2f\cos {(b^*\tau _c)}}=\dfrac{a\cos {(b^*\tau _c)}-c\cos {(2b^*\tau _c)}-d\cos {(3b^*\tau _c)}-e}{2g\cos {(b^*\tau _c)}+b}, \end{aligned}$$

where $a=\nu _3{b^*}^2-\nu _5$, $b=\nu _4{b^*}$, $c=\nu _7$, $d=\nu _8$, $e=\nu _9-\nu _1{b^*}^2$, and $f={b^*}^3-\nu _2{b^*}$. Then, by comparing the calculated value of $\sin (b^* \tau _c)$, we arrive at the following result

16 $$\begin{aligned} \cos (b^*\tau _c)[8m_1\cos ^3(b^*\tau _c)-(4q_1+2t_1)\cos ^2(b^*\tau _c) -(6m_1+2p_1+s_1)\cos (b^*\tau _c)-(r_1-t_1-3q_1)]=0. \end{aligned}$$

where,

$$\begin{aligned} m_1=& \,{} d_1f_1, r_1=2c_1g_1+b_1d_1-2a_1b_1-2e_1f_1, s_1=2d_1g_1-2a_1g_1+2a_1f_1,\\ t_1=&\, {} 2e_1g_1-2c_1f_1, p_1=b_1e_1+b_1c_1, q_1=b_1d_1, \end{aligned}$$

with

$$\begin{aligned} a_1=&\, {} b^{*2}\nu _3-\nu _5, b_1=b^*\nu _4, c_1=\nu _7,\\ d_1=&\, {} \nu _8, e_1=\nu _9-b^{*2}\nu _1, f_1=b^{*3}-b^*\nu _2. \end{aligned}$$

After substituting all the relevant terms as mentioned above, the above equation can be rewritten as:

$$\begin{aligned} cos(b^*\tau _c)[\xi _1cos^3(b^*\tau _c)-\xi _2cos^2(b^*\tau _c)-\xi _3cos(b^*\tau _c)+\xi _4]=0, \end{aligned}$$

where

$$\begin{aligned} \xi _1=&\, {} 8b^*\nu _8(b^{*2}-\nu _2),\\ \xi _2=& {} -4b^*[(\nu _1\nu _6+\nu _7)b^{*2}-(\nu _2\nu _7+\nu _4\nu _8+\nu _6\nu _9)],\\ \xi _3=&\, {} 2b^*[b^{*4}\nu _3+(3\nu _3-\nu _1\nu _4-\nu _2\nu _3-\nu _3\nu _6-\nu _5)b^{*2}\\{}\, & {} +(\nu _2\nu _5+\nu _4\nu _7+\nu _4\nu _3+\nu _5\nu _6+\nu _6\nu _8-3\nu _2\nu _8)],\\ \xi _4=&\, {} -b^*[2b^{*4}\nu _1-2(\nu _1\nu _2+\nu _3\nu _4+\nu _9-\nu _1\nu _6-\nu _7)b^{*2}\\{} & {} \,-(2\nu _2\nu _7+3\nu _4\nu _8+2\nu _6\nu _9-2\nu _2\nu _9-\nu _4\nu _8-2\nu _4\nu _5-2\nu _6\nu _7)]. \end{aligned}$$

Equation (16) brings forth one of the solutions as $\cos (b^*\tau _c)=0$. Therefore, we need to determine the value of $b^*$ for which the relation $\cos (b^* \tau _c)=0$ holds true. We can use this relationship to calculate the critical value $\tau _c$, at which Hopf bifurcation occurs. Therefore, the value of $\tau$ obtained from this relationship can be considered as the value of $\tau _c$.

**Figure 9:**
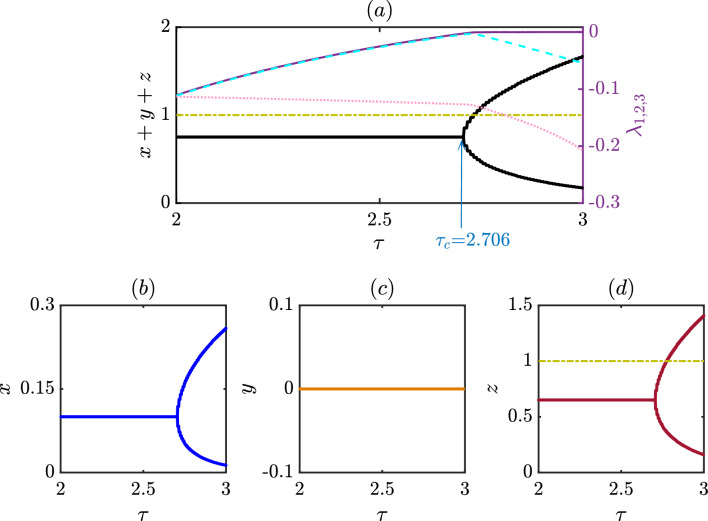
Verification of the analytically obtained value of the critical time delay $\tau _c$: (**a**) Demonstrates the bifurcation diagram and three Lyapunov exponents of the delay model by varying the delay parameter $\tau$ in the range [2, 3]. We use the following parameter values: $\xi = 0.40$, $\beta = 1.50$, $\delta = 0.56$, $\sigma _{1} = 1.20$, $\sigma _{2} = 1.70$, and $\sigma _{3} = 1.0$. For the chosen parameter values the system undergoes a Hopf bifurcation at the critical point $\tau _c = 2.706$, that matches perfectly with the analytically derived $\tau$ critical value. The largest Lyapunov exponent $\lambda _1$ shown with solid purple line becomes zero at the bifurcation point and remains unchanged beyond the critical point. The second largest Lyapunov exponent $\lambda _2$ (cyan dashed line) touches zero at the bifurcation point and remains negative for other values of $\tau$, and the third exponent $\lambda _3$ (pink dotted line) remains negative throughout the range of $\tau$. Beyond a certain value of $\tau$ the system exhibits overcrowded solution as the total population $x+y+z$ exceeds the maximum value 1. A green horizontal dash dotted line is plotted to mark the boundary of the overcrowded solution from that of the bounded solution. (**b**–**d**) Showcase the effect of delay $\tau$ on each of the population variables. Punishers are unable to survive for the chosen parameter values even though they receive most benefit from the free space compared to the cooperators and defectors. We choose initial fractional quantities for the non-delayed variables to be (0.1, 0.2, 0.5) and for the delayed variables to be (0.3, 0.3, 0.3).

By modifying Eq. (14), we can obtain a sixth-degree polynomial in terms of $b^*$. Substituting the value of $\sin (b^* \tau _c)|_{\cos (b^* \tau _c)=0}$ from Eq. (15), we can derive an equation in terms of $b^*$, which is sufficient to calculate $\tau _c$. The resulting sixth-degree equation is

17 $$\begin{aligned} b^{*6}+\eta _1b^{*4}+\eta _2b^{*2}+\eta _3=0, \end{aligned}$$

where

$$\begin{aligned} \eta _1=&\, {} \nu _1^2+\dfrac{2\nu _1\nu _3\nu _6}{\nu _4}-2\nu _2-\nu _3^2,\\ \eta _2=& \,{} \dfrac{2\nu _6}{\nu _4}\{\nu _3(\nu _7-\nu _9)+\nu _1\nu _8-\nu _1\nu _5\}+2\nu _1\nu _7-(2\nu _1\nu _9+\nu _4^2+\nu _6^2+2\nu _3\nu _8-\nu _2^2-2\nu _3\nu _5),\\ \eta _3=&\, {} \nu _9^2+2\nu _5\nu _8-\nu _5^2-\nu _7^2-\nu _8^2+\dfrac{2\nu _6}{\nu _4}(\nu _7-\nu _9)(\nu _8-\nu _5)+2\nu _7(\nu _7-\nu _9). \end{aligned}$$

The roots of Eq. (17) are the six values of $b^*$ such that the relation for the critical value of $\tau$ holds, i.e., $cos(b^*\tau _c)=0$, which implies $(b^*\tau _c)=(2n+1)\dfrac{\pi }{2}$. For $n=0$, the critical value $\tau _c$ can be calculated as $\tau _c=\dfrac{\pi }{2b^*}$, where the value of the required $b^*$ can be calculated from Eq. (17). Note that one can obtain at most six different real values for $b^*$. Only one of these real roots provides us with our desired critical value of the delay parameter at the Hopf bifurcation.

### Validation of analytical finding

In Fig. 3, we observe the occurrence of Hopf bifurcation, where a stable equilibrium point becomes unstable, giving rise to a periodic solution at $\tau =8.98$. This critical point is consistent with the analytically derived $\tau _c$.

Furthermore, in Fig. 9, we demonstrate the bifurcation diagram of our system (6) by varying the delay parameter $\tau$ within the range [2, 3], using a fixed step length of 0.002, with $\xi =0.40$, $\beta =1.50$, $\delta =0.56$, $\sigma _{1}=1.20$, $\sigma _{2}=1.70$, and $\sigma _{3}=1.0$. Despite the fact that free space favors punishers due to $\sigma _2>\sigma _1>\sigma _3$, punishers eventually become extinct in the long run (as seen in Fig. 9c). In the steady-state regime, defectors can dominate cooperators, even though they are less favored by the free space (as seen in Figs. 9b,d). However, this steady-state loses its stability at $\tau =2.706$, resulting in a limit cycle oscillation. The occurrence of Hopf bifurcation at this point in the system parameter space confirms our analytically derived $\tau _c=2.706$. The maximum Lyapunov exponent $\lambda _1$, shown with solid purple line in subfigure (a), remains at zero beyond this bifurcation point, confirming the periodic oscillation of the system in the range studied. Additionally, the second-largest Lyapunov exponent $\lambda _2$, shown with cyan dashed line, touches zero at this bifurcation point, further validating our bifurcation diagram. However, beyond a certain value of the delay parameter $\tau$, the system’s solution becomes overcrowded, as the values of $(x+y+z)$ surpass the upper bound of 1. We highlight this ecologically feasible range by plotting a horizontal dash-dotted line in subfigures (a) and (d), and solutions below this line remain ecologically meaningful and interpretable.

## Discussion

Eco-evolutionary dynamics investigates how ecological and evolutionary processes interact and influence each other over time. It involves understanding the feedback loops between ecological and evolutionary processes and how they shape the dynamics of populations and communities. Ecological processes such as competition, predation, and resource availability can drive natural selection and thus influence the evolution of traits in populations. Conversely, the traits and genetic diversity within populations can shape ecological processes and affect the structure and functioning of communities. Eco-evolutionary dynamics can have important implications for conservation biology, as changes in ecological processes or environmental conditions can alter the direction or rate of evolution and vice versa. For example, human-induced environmental changes such as habitat fragmentation or climate change can alter the selective pressures on populations, leading to changes in their evolutionary trajectory. Overall, understanding the complex interplay between ecological and evolutionary processes is crucial for understanding the functioning and resilience of ecosystems in the face of environmental change.

Our present study examines the interactions and long-term effects between ecological and evolutionary processes by considering a classic game, viz. the prisoner’s dilemma. The prisoner’s dilemma is often used as a metaphor for situations in which two individuals or groups must choose between cooperation and competition and in which self-interest can lead to a suboptimal outcome for both parties. It has important applications in economics, political science, and evolutionary biology. Our study suggests free space being an ecological variable, can significantly influence the prisoner’s dilemma game’s evolutionary dynamics. Free space here acts as an individual that benefits another individual at a cost. They lose their fitness by helping others without any expectation of reward. Although this form of philanthropic activity may seem to be foolish and may raise a question on the evolutionary basis of altruism among researchers, one can still observe such altruistic behavior in a variety of forms, such as donating to charity, volunteering time to help others, or even sacrificing one’s own life to save another. Altruism can also occur in non-human species, such as when a mother animal risks her safety to protect her offspring. We incorporate this vital aspect of social behavior which has important implications for fields such as psychology, sociology, and evolutionary biology. In contrast, punishment is an effective strategy to discourage individuals from engaging in undesirable behavior by making the cost of that behavior exceed the benefit. Punishment can provide a sense of justice or closure for those harmed by the conduct and can prevent people or groups from behaving in ways that are undesirable or unacceptable. To incorporate punishment into our game, we introduce a fraction of punishers. This approach, combined with the classical prisoner’s dilemma game and two additional strategies, helps us to understand how ecological and evolutionary processes interact and impact population and community dynamics.

However, despite making progress, we have limited knowledge of how time lag affects eco-evolutionary game dynamics. The study of dynamical systems with delay is an active research area in mathematics, physics, and engineering. It has practical applications in fields such as control theory, signal processing, and neuroscience. Through numerical simulations, we find that delay can lead to oscillation in our system, which is impossible in non-delayed cases with those sets of parameter values. The evolution of strategies depends on several factors, such as the resources punishers use to fine defectors and the charitable contribution of free space. The survival of players using different strategies also depends on the initial fraction of the population. The system can settle into various stable states, and understanding multistability can provide valuable insights into the complex behavior of our system. We derive all steady states and calculate the point of Hopf bifurcation, where the steady state loses its stability, and delay causes the system to undergo periodic oscillations. This periodic attractor enables each strategy to dominate the other in a cyclical manner under suitable circumstances. Although defection appears to be the rational choice in the classical prisoner’s dilemma game, the intricate interactions between ecological and evolutionary processes prevent any strategy from dominating the others in the long run. Our proposed eco-evolutionary model can behave like a three-species cyclic dominance system for an appropriate selection of parameters, with each species dominating and being outcompeted by the next. Following the Hopf bifurcation, the system may undergo a series of period-doubling bifurcations as the parameter value increases, resulting in a successive doubling of the period of oscillation and the emergence of chaotic behavior. However, this can lead to overcrowding and limit the biological implications of our study. Our study also has several limitations worth exploring further. Our study uses paired communications to characterize group interactions and is conducted for the prisoner’s dilemma game. Investigating the impact of higher-order interactions^81,82^ and how the outcome varies for other evolutionary games^83,84^ might be fascinating. In conclusion, our analysis provides valuable insights into the complex eco-evolutionary dynamics and contributes to a better understanding of group decision-making and the emergence of moral behavior in multidimensional social systems. We hope that our study will inspire further research in this area and facilitate the development of effective strategies for managing and conserving ecological communities.

## Data Availability

The datasets used and/or analyzed during the current study available from the corresponding author on reasonable request.
